# High-precision binary trait association on phylogenetic trees

**DOI:** 10.1099/mgen.0.001791

**Published:** 2026-07-31

**Authors:** Ishaq O. Balogun, Christopher P. Mancuso, Tami D. Lieberman

**Affiliations:** 1 Institute for Medical Engineering and Sciences, Massachusetts Institute of Technology, Cambridge, MA 02142, USA; 2 Department of Civil and Environmental Engineering, Massachusetts Institute of Technology, Cambridge, MA 02142, USA

**Keywords:** binary trait evolution, epistasis, gene–gene interactions, microbial genome-wide association study (GWAS), pangenome, phylogenetic association

## Abstract

Traditional methods for identifying associations between genomic features and traits, or between pairs of genomic traits, struggle when applied to bacterial genomes. While several microbial genome-wide association study (mGWAS) methods have been developed to account for the fact that genome-wide linkage in bacteria creates strong evolutionary-induced associations, these methods have high false discovery rates or lack statistical power, have poor performance on negative interactions and face computational limits at the scale required for pangenome-wide study of gene–gene interactions. Here, we present Simulation-based Phylogenetic iNteraction Inference (SimPhyNI), a computationally optimized framework for efficient and rigorous mGWAS studies. SimPhyNI builds null co-occurrence distributions by independently simulating traits using phylogenetically informed parameters, novelly including time to first event. The constrained variation in these simulations, combined with log odds ratio scoring for comparing across traits, robustly identifies both positive and negative associations. Using synthetic datasets mimicking both gene–gene and gene–trait associations, we demonstrate that SimPhyNI achieves high precision and recall for both positive and negative interactions. We demonstrate SimPhyNI’s utility by detecting interactions between phage defence systems in *Escherichia coli* and gene–gene interactions across the entire *E. coli* pangenome (>9 million tests). Though developed here for binary traits, SimPhyNI’s design supports extension to multi-state and continuous traits using generalized models of stochastic simulation. SimPhyNI’s performance and scalability enable genome-wide discovery of genetic interactions that drive microbial function, ecology and disease.

**Impact Statement**

Understanding how bacterial genes associate with traits and with one another is essential for predicting disease outcomes, antibiotic resistance and future evolution. However, identifying these interactions is challenging because shared ancestry creates spurious correlations. Simulation-based Phylogenetic iNteraction Inference (SimPhyNI) overcomes this through an ancestry-informed statistical simulation process, achieving near-zero false positive rates while maintaining computational efficiency for large scale analyses. This efficiency enables systematic mapping of gene–gene interaction networks across large datasets containing thousands of genes and genomes. As microbial genomic datasets continue to expand, SimPhyNI’s scalability and precision will accelerate discovery of the mechanistic principles underlying infectious disease, microbiome function and microbial evolution and ecology.

## Data Summary

Simulation-based Phylogenetic iNteraction Inference is publicly available at https://github.com/jpeyemi/SimPhyNI, and code for related benchmarking, validation and biological analyses are available at doi.org/10.5281/zenodo.18024873. The neighbour-joining phylogenetic tree and phage defence system annotations used in this study were obtained from Wu *et al*. [25]. Representative species genomes and their corresponding phylogenetic trees were obtained from the PanX database (https://pangenome.org).

## Introduction

Identifying associations is a critical step in genomic analysis – both for understanding how genetic variation affects traits and how interactions between genomic loci (epistasis) shape evolution. In particular, each bacterial genome contains only a fraction of the genes in its species pangenome [1–3], and understanding the consequences of this variation for health and the gene–gene interactions that constrain evolutionary trajectories are foundational challenges in microbiology [4, 5].

However, traditional genome-wide association study (GWAS), a standard approach for identifying genetic associations in eukaryotes [6], is not easily applied to bacterial genomes [7]. Microbes reproduce asexually, with limited recombination to break up genes on distant parts of the genome; as a consequence, variants across the genome can be inherited together and perfectly correlate with one another as an artefact of evolutionary relatedness. This non-independence breaks the assumptions of traditional statistical tests and makes it difficult to define genomic variants and traits that are associated due to true biological interactions [7, 8].

**Fig. 1.**
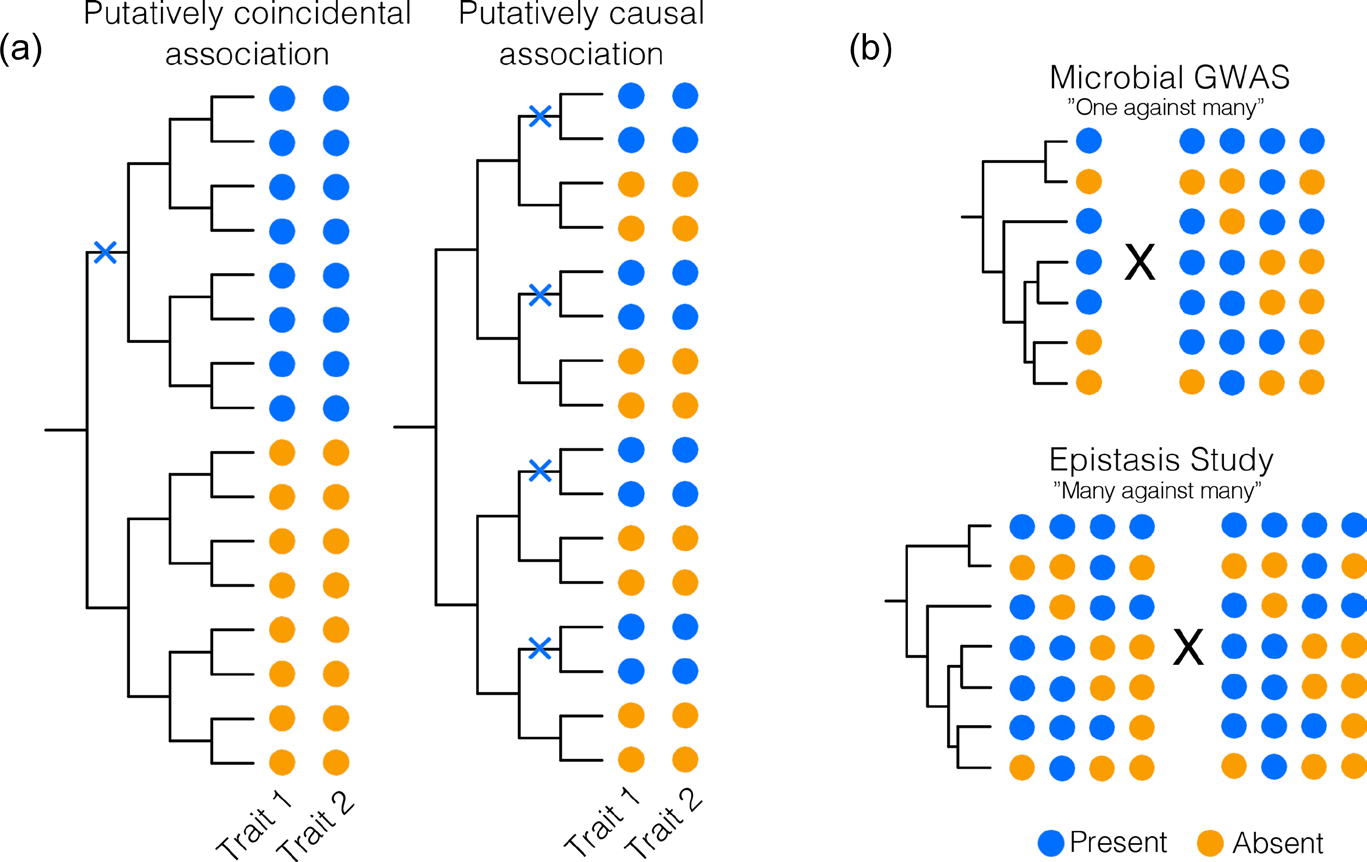
Microbial genome-wide association studies (mGWASs) and epistasis tools aim to correct for phylogenetic signal. (**a**) Two evolutionary scenarios showcasing the same number of trait co-occurrences but with different assortment on the same phylogenetic tree. Inferred locations of co-incident trait events are labelled with Xs. While both scenarios are numerically identical for traditional statistical methods that do not consider evolutionary relationships, convergent patterns of co-occurrence across district evolutionary events provide more evidence of a direct relationship between two traits. (**b**) mGWAS (top) uses a one against many approach to test a single phenotype against an array of genotypic information, yielding a number of comparisons linear with the number of genotypes. In contrast, epistasis studies (bottom) compare sets of genotypic information in a many-against-many fashion, resulting in a number of comparisons that grows quadratically with the number of genotypes.

Identifying meaningful biological interactions in microbial genomes requires identifying cases where trait–variant or variant–variant pairs are frequently found together as a result of multiple independent evolutionary events. Traditional statistical methods that ignore phylogenetic relationships cannot differentiate between scenarios with identical co-occurrence counts but different evolutionary histories. For example, a single horizontal gene transfer (HGT) event followed by vertical inheritance may produce the same numerical association as multiple independent events across distant lineages, but the latter pattern provides stronger evidence of mechanistic synergy driving gene co-occurrence (Fig. 1a). However, reconstructing these evolutionary histories is challenging. Ancient events may be obscured by subsequent losses, HGTs and phylogenetic uncertainty. Methods must therefore balance the need to account for shared ancestry while remaining robust to incomplete evolutionary information.

Although efforts have been made to incorporate phylogenetic information into association studies [8–10], current methods for microbial GWAS (mGWAS) face trade-offs between statistical power, false positive control and computational scalability [7, 11]. Pagel’s correlation method [9] is well established as a standard for the association testing of binary traits; however, it requires fitting computationally expensive statistical models for the likelihood of independent vs dependent evolution. Modern mGWAS tools such as FaST-LMM [10], TreeWAS [8] and Scoary [12, 13] employ more computationally efficient and widely applicable options but do not have strong separation between false positives and true associations, creating a trade-off between stringent false positive control and the recall of interactions. Epistasis studies are particularly challenging for mGWAS studies (Fig. 1b), requiring additional statistical power and computational efficiency to evaluate the sheer number of possible pairwise interactions.

**Fig. 2.**
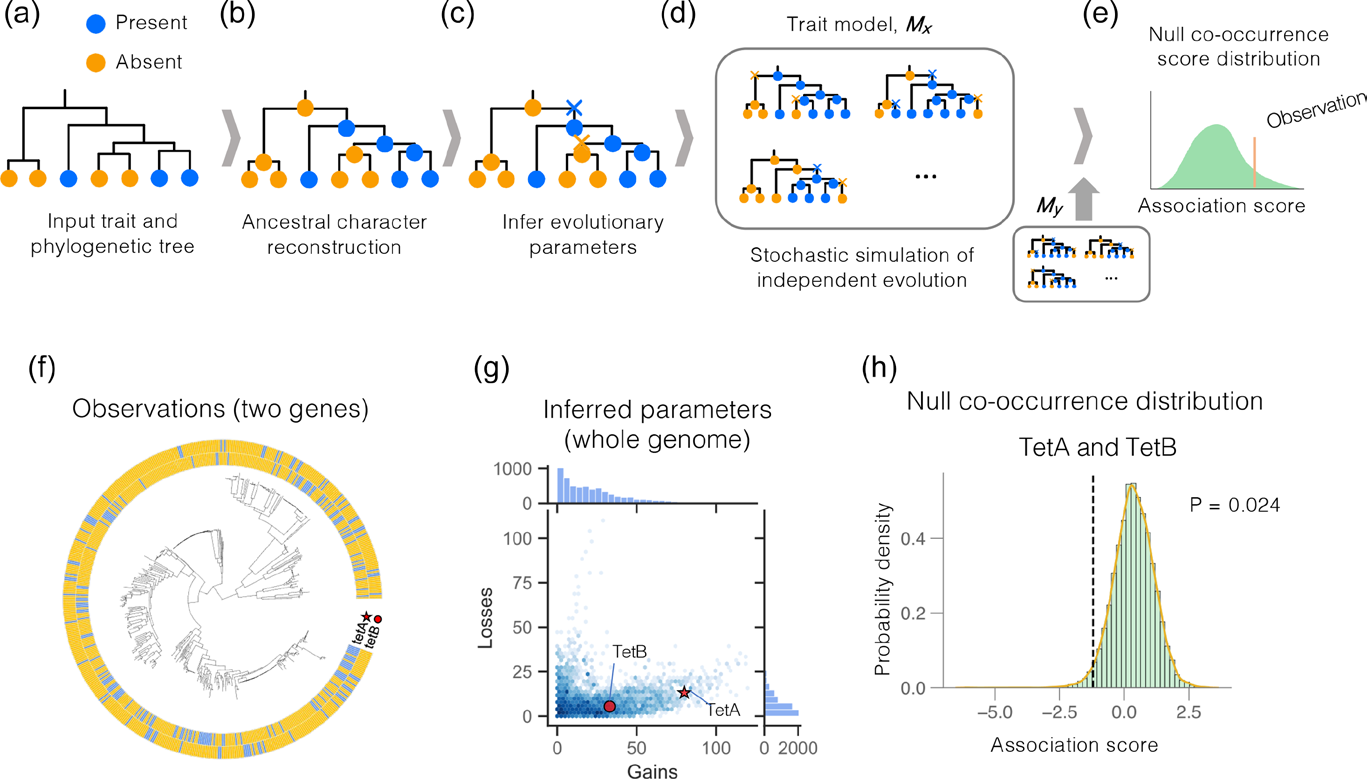
SimPhyNI is a trait association method for mGWAS and epistasis studies. (**a**) SimPhyNI begins with an input phylogeny and annotations of trait presence (blue) or absence (orange) on the tips of the tree. (**b**) Ancestral character reconstruction of the input tree is then performed, using a maximum likelihood approach to infer the presence or absence at each node (Methods). (**c**) Evolutionary parameters of trait gain and loss are inferred using a Markov chain learning process (gain rate, loss rate and time to first event; Methods). Orange and blue Xs represent inferred state transitions. (**d**) SimPhyNI then performs many stochastic simulations of a trait under a model of independent evolution using the inferred parameters, resulting in a trait matrix Mx (Methods). (**e**) This is compared with a trait matrix for the second trait, My, to generate a null co-occurrence score distribution which is compared to the co-occurrence score observed (Methods). (**f–h**) An example of two interacting genes. (**f**) Observed presence and absence of tetracycline efflux pump genes, TetA and TetB, on a 500-isolate *E. coli* phylogenetic tree. (**g**) Distribution of inferred gains and losses for 4,333 *E. coli* accessory genes from discretized maximum marginal probability ancestral character reconstruction, with TetA and TetB labelled. (**h**) Histogram of the final statistical readout of TetA and TetB, comparing the observed co-occurrence score with the simulated null distribution. The orange line indicates the approximated distribution (Methods) used for *P*-value computation.

Here, we present Simulation-based Phylogenetic iNteraction Inference (SimPhyNI), a computationally efficient and robust tool to evaluate associations of binary traits across microbial genomes. SimPhyNI uses the phylogenetic tree to parameterize each trait’s evolutionary history – including rates of gain, loss and emergence timing – and then simulates their independent evolution to estimate expected co-occurrence patterns under a null model of no interaction (Fig. 2). SimPhyNI operates on binary traits (gene presence/absence, single nucleotide variants (SNVs) and binarized phenotypes), providing sufficient statistical power with hundreds to thousands of genomes – the current practical limit for rigorous phylogenetic reconstruction of diverse sets of genomes of the same bacterial species [14].

SimPhyNI achieves superior performance through two key innovations: simulations that account for uncertainty in deep ancestral branches, improving our null models for trait evolution, and a prevalence-aware scoring function, improving statistical power across variable tree topologies. For computational efficiency, we employ kernel density estimation (KDE) to approximate null distributions, reducing the number of simulations needed to evaluate traits while maintaining statistical power. Together, these optimizations enable SimPhyNI to scale to millions of pairwise interactions without sacrificing statistical rigour or recall.

Using synthetic datasets, we demonstrate that SimPhyNI recovers both positive and negative associations with high accuracy while maintaining exceptionally low false positive rates (FPRs). We apply SimPhyNI in both a highly directed search for interactions between phage defence systems in *Escherichia coli* and a comprehensive pangenome analysis spanning 9.4 million pairwise associations. We further demonstrate SimPhyNI’s applicability to larger genome collections and other binary-encodable variant types, including SNVs and indels, through an analysis of 195,282 variants across 59,353 *Mycobacterium tuberculosis* isolates. As microbial genomic datasets continue to expand, SimPhyNI’s scalability and accuracy will accelerate discovery of the evolutionary constraints and functional dependencies that shape bacterial adaptation and pathogenesis.

## Methods

### SimPhyNI implementation

#### Reconstruction of phylogenetic tree

A well-formed phylogenetic tree is necessary for SimPhyNI, as parameter estimation and trait model generation both rely on phylogenetic structure and evolutionary distance between samples. For this purpose, maximum likelihood tree estimation is recommended for accurate branch lengths, though neighbour joining methods can be used when the sample size is prohibitively large. While tree construction is not included in the primary SimPhyNI package, we have also developed SimPhyNI-Prelude (https://github.com/jpeyemi/SimPhyNI-Prelude), a pipeline with modules for genome annotation, phylogenetic reconstruction, pangenome graph building, SimPhyNI and downstream colocation analysis for gene–gene association studies.

#### Trait parameter estimation

The first step of SimPhyNI is to estimate the parameters of past evolution: gain rate, loss rate, root state and time to first event. These are obtained by performing ancestral character reconstruction (ACR) independently for each trait, starting with a rooted phylogenetic tree and tip annotations (Fig. 2a, b). We use a custom implementation of the marginal posterior probability approximation (MPPA) method under the F81 character evolution model as used in PastML [15], which yields state probabilities at each internal node used for downstream rate calculation. MPPA is more accurate than maximum parsimony methods favoured by other simulation-based GWAS approaches [8], but more computationally costly [15]. To recover efficiency, we use a closed-form analytical solution of the F81 character model for binary states [16] and golden-section search for gradient-free parameter optimization. Both reduce all computation to scalar operations, enabling efficient compilation to machine code using Numba (v0.62.0), scaling ACR to hundreds of thousands of traits (Fig. S16, available in the online Supplementary Material).

To turn ancestral states into parameters that describe the rates of gain and loss for each trait, each phylogenetic tree is traversed from root to tip using the MPPA computed during ACR (Fig. 2c). First, the trait state at the root of the phylogeny (root state) is calculated as the state with marginal probability >0.5. Then, evolutionary distance from the root to the first inferred emergence of each trait (*d*_emergence_(𝑥), e.g. first gain or first loss) is calculated, determined by the first node with probability of the non-root state >0.5. Transition rates are computed by normalizing the total observed state changes against the total available branch length, expressed in arbitrary evolutionary distance units. Thus, the gain rate is defined as


$$\lambda =\frac{\sum_{b\in B}\max\left(0,\;P(1|c_{b})-P(1|p_{b})\right)}{\sum_{b\in B}P(0|p_{b})\;l_{b}}$$

and the loss rate as


$$\mu =\frac{\sum_{b\in B}\max(0,P(1|p_{b})-P(1|c_{b}))}{\sum_{b\in B}P(1|p_{b})\;l_{b}}$$

where *p_b_* and *c_b_* are the parent and child of a given branch *b* and *P*(1∣⋅) is the marginal posterior probability of state 1.

To avoid artificially deflating transition rates due to long branches with hidden transitions, long branches are truncated using an interquartile range (IQR) based method applied in log_10_ space. Branches longer than 10*^Q^*^3+0.5×IQR^ – where IQR = *Q*3 − *Q*1 is computed in log_10_ space – are truncated to this length for eligible branch length calculation. In practice, this truncation affects fewer than 1% of branches in the *E. coli* application tree and a mean of 3.2% across benchmarking trees. Both this IQR threshold and the use of *d*_emergence_(𝑥) were tested through iteratively parameterizing and simulating traits to ensure minimal measured drift from the original seed across trait diagnostic metrics (prevalence, Fitch parsimony, phylogenetic D and mean pairwise distance; see Fig. S5).

#### Independent trait model

SimPhyNI then simulates the independent evolution of each trait along the phylogeny as a stochastic process, leveraging the learnt parameters from the previous step (Fig. 2d). For each trait 𝑥, this procedure generates a trait model: a set of simulated trait occurrences, represented as a matrix


$$X\in \{0,1{\}}^{N\times t}$$

where *N* is the number of terminal nodes (isolates) and *t* is the number of independent simulations.

Simulations are generated using a Markov process that traverses the phylogeny from root to tips. The root state is initialized according to the ACR inferred state. For each branch *b* of length *l_b_*, state transitions are modelled as a Poisson process with transitions sampled according to gain rate, *λ*, and loss rate, *μ*. If the parent state is absent, the probability of a gain is


$$p_{gain,b}(x)=1-\exp\left(-\lambda (x)\;l_{b}\right)$$

and if the parent state is present, the probability of a loss is


$$p_{loss,b}(x)=1-\exp\left(-\mu (x)\;l_{b}\right)$$

In order to prevent trait emergence at much deeper branches of the tree than empirically observed, evolutionary events are not permitted before nodes that have a cumulative distance from the root less than the emergence threshold *d*_emergence_(𝑥) (see Table S1 for overviews of tested variations of this implementation). The results are then stored as a single column matrix *X*. This process is repeated 50≤*t*≤1,000 times, depending on whether a coarse or highly resolved approximation of downstream probability distributions is required (see Fig. S2 for number of comparisons required).

#### Computing pairwise co-occurrence null distributions

For each pair of traits, a null distribution of trait co-occurrence is computed by comparing the two trait model matrices (Fig. 2e). Let *x* and *y* be two traits of interest, with simulated trait matrices


$$X,Y\in \{0,1{\}}^{N\times t}$$

where *N* is the number of terminal nodes and *t* is the number of independent simulations of the trait.

We then consider sets of columns between these two matrices. For each pair of simulation columns (*X_i_*, *Y_j_*), we compute a co-occurrence score using the log odds ratio of tip annotations:


$$Score(X_{i},Y_{j})=\log\left(\frac{\left(a_{ij}+\epsilon )(d_{ij}+\epsilon \right)}{\left(b_{ij}+\epsilon )(c_{ij}+\epsilon \right)}\right)$$

where

$a_{ij}=\sum_{n=1}^{N}X_{i,n}Y_{j,n}$ (both traits present)

$b_{ij}=\sum_{n=1}^{N}X_{i,n}\left(1-Y_{j,n}\right)$ (only *x* present)

$c_{ij}=\sum_{n=1}^{N}\left(1-X_{i,n}\right)Y_{j,n}$ (only *y* present)

$d_{ij}=\sum_{n=1}^{N}\left(1-X_{i,n}\right)\left(1-Y_{j,n}\right)$ (neither trait present)

ϵ=1, (small discrete constant to avoid undefined log values and extreme edge behaviour)

This log odds scoring function was chosen after comparison to other scoring metrics (see Fig. S1 and Table S2).

Exhaustively computing scores across all pairs of columns creates a discrete null set of size *t*^2^.


$$S_{null}^{\left(x,y\right)}=\left\{Score(X_{i},Y_{j}))|1\leq i,j\leq t\right\}$$

Gaussian KDE is then used to approximate a continuous null distribution of co-occurrence scores from the discrete distribution.


$$f_{null}^{\left(x,y\right)}=KDE\;\left(S_{null}^{\left(x,y\right)}\right)$$

Using estimated null distributions reduces the number of trait simulations required, increasing computational efficiency while preserving statistical power and enabling higher resolution *P*-values. Silverman’s selection method is applied to determine optimal KDE bandwidths, as observed null distributions are approximately normal (Fig. S3; though this approach also handles multimodality sometimes seen, Fig. S4). Notably, taking the logarithm of the odds ratio (rather than using the raw odds ratios) is critical for KDE performance, as Gaussian KDE requires distributions without high skew.

#### Calculating significance and effect size

Using the approximated continuous null distribution for each set of traits, $f_{null}^{\left(x,y\right)}$, we perform statistical hypothesis testing to identify significant trait-trait associations. For each observed co-occurrence score, an empirical *P*-value is computed using the approximated distribution (Fig. 2e; full example traits, Fig. 2f–h).

To control for false positives, we apply the Benjamini–Yekutieli (BY) procedure [17] rather than the more common Benjamini–Hochberg (BH) [18]. BH is guaranteed to control the false discovery rate (FDR) only under independence or positive regression dependence on a subset[17], which cannot be assumed here, as naive *P*-values are correlated through co-inheritance, functional co-occurrence and shared phylogenetic history. Under such dependence, BH can both fail to control FDR in expectation and produce highly variable rejection sets in which individual experiments yield many false positives at once [19–21]. BY trades modest power for explicit FDR control under arbitrary dependence – a trade-off we accept given the scale and dependence structure of genome-wide screens.

Effect sizes were computed as the normalized difference between observed and expected co-occurrence scores:


$$Effect\;Size=\frac{X_{obs}-Median\;\left(f_{null}^{\left(x,y\right)}\right)}{IQR\;\left(f_{null}^{\left(x,y\right)}\right)/1.349}$$

This calculation used median and IQR with a scaling factor in order to resemble a Z-score for normal null distributions. However, moderate skew and multimodality have been observed in empirical null distributions (Figs S3 and S4), so median and IQR were selected to reduce the sensitivity of effect size to outlying data.

### Synthetic dataset construction

Synthetic datasets with varying tree topologies and effect sizes were constructed for the validation and benchmarking of SimPhyNI against existing mGWAS methods. Each dataset consists of a single 500-strain synthetic phylogenetic tree generated using msprime [22] with default parameters and a collection of synthetic interacting trait pairs. For each dataset, we simulated 300 positively correlated pairs, 300 negatively correlated pairs and randomly sampled 3,000 pairs of traits with no enumerated interaction parameter to be our null set; these unbalanced classes emulate the expectation that most gene pairs do not interact.

Synthetic trait pairs were generated using a four-state continuous-time Markov chain, transitioning between states {00, 01, 10, 11} representing all joint presence/absence combinations of two binary traits, with only single-bit transitions permitted [9, 23].

For each interacting trait pair, we (1) sampled two independent traits with prevalence, gain and loss rates and phylogenetic distributions designed to match empirical data and (2) introduced an interaction parameter that controls the strength and direction of association.

First, each trait was parameterized with an equilibrium prevalence, *π* = *λ* / (*λ* + *μ*), and a total transition rate, Λ, sampled independently from empirical distributions of *π* and Λ computed from the *E. coli* accessory genome. For this process, we used two methods of computing Λ, sampled with equal probability, that differ in how they treat the uncertainty of latent ancestral states. One method used MPPA-derived rates, Λ = *λ* + *μ* (see Trait parameter estimation), which integrates the flow of probability across all ancestral states and culminates in higher Λ values (high transition model). The other used discretized states to compute total rate from discrete gain and loss counts, *n_G_* and *n_L_*, normalized by the reference tree total branch length, *B*_ref_. This formulation, $\Lambda =\frac{n_{G}+n_{L}}{B_{ref}},$ excludes uncertain states from rate calculation, yielding lower Λ values (low transition model). These two estimates were developed to conceptually and empirically mirror the range of highly mobile to highly conserved accessory genes. When simulating individual traits with these parameterizations on the *E. coli* phylogenetic tree, both models reproduce the empirical prevalence distributions by design. Jointly, they reproduce both the central tendency and the full range of *E. coli* empirical phylogenetic D-statistic and Fitch parsimony distributions, whereas either model alone spans only part of this range (Fig. S6).

To apply sampled values to new tree topologies, Λ was rescaled by the ratio of IQR-filtered total branch lengths (see Trait parameter estimation) between the reference tree and the target simulated tree, $\Lambda_{scaled}=\Lambda \cdot \frac{B_{ref}}{B_{sim}},$ while *π* was left unchanged, as it is a dimensionless ratio of rates.

The interaction parameter, *I*, encodes magnitude and direction of interactions as a target odds ratio, OR = 10^2^*^I^*, with *I* > 0, *I* < 0 and *I* = 0 yielding positive associations, negative associations and independent evolution, respectively. Given *π*_1_, *π*_2_ and OR, the four-state stationary distribution, s = [*p*00, *p*10, *p*01, *p*11], was solved analytically via the following quadratic:


$$(1-OR)p_{11}^{2}+\left(1-\pi_{1}-\pi_{2}+OR(\pi_{1}+\pi_{2})\right)p_{11}-OR\;\pi_{1}\pi_{2}=0$$

The physically valid root in $\left(\max(0,\pi_{1}+\pi_{2}-1),\;\min(\pi_{1},\pi_{2})\right)$ was selected; for OR > 1, the larger root was taken, and for OR < 1, the smaller root was taken.

A rate matrix *Q* was then constructed with *Q*[*i*, *j*] = *k* · *s_j_* for each connected state pair (*i*, *j*), guaranteeing that s – and by extension the target prevalences and OR – are exact properties of the chain. The scale, *k*, was set to equal the geometric mean of the scaled total rates, $\Lambda_{mean}=\sqrt{\Lambda_{scaled,1}\cdot \Lambda_{scaled,2}}$. Trait pairs were then simulated along each tree with a root state drawn from s. Phylogenetic structure and Λ_mean_ determine how close trait pairs approach the target OR (Fig. S7).

Using 11 datasets (each with 1 tree and 3,600 synthetic trait pairs) at tested interaction strengths I∈{−2,−1,−0.7,−0.5,−0.15,0,0.15,0.5,0.7,1,2}, the distribution of SimPhyNI-measured effect sizes was compared to the effect size distribution from SimPhyNI analysis of the *E. coli* pangenome. *I* = −1 for negative associations and *I* = 1 for positive associations best mirrored effect sizes from the empirical data (Fig. S8) and were selected as the standard interaction parameters for method evaluation. All methods were evaluated using BY FDR correction at a threshold of 0.01.

### Benchmarking against other methods

**Fig. 3.**
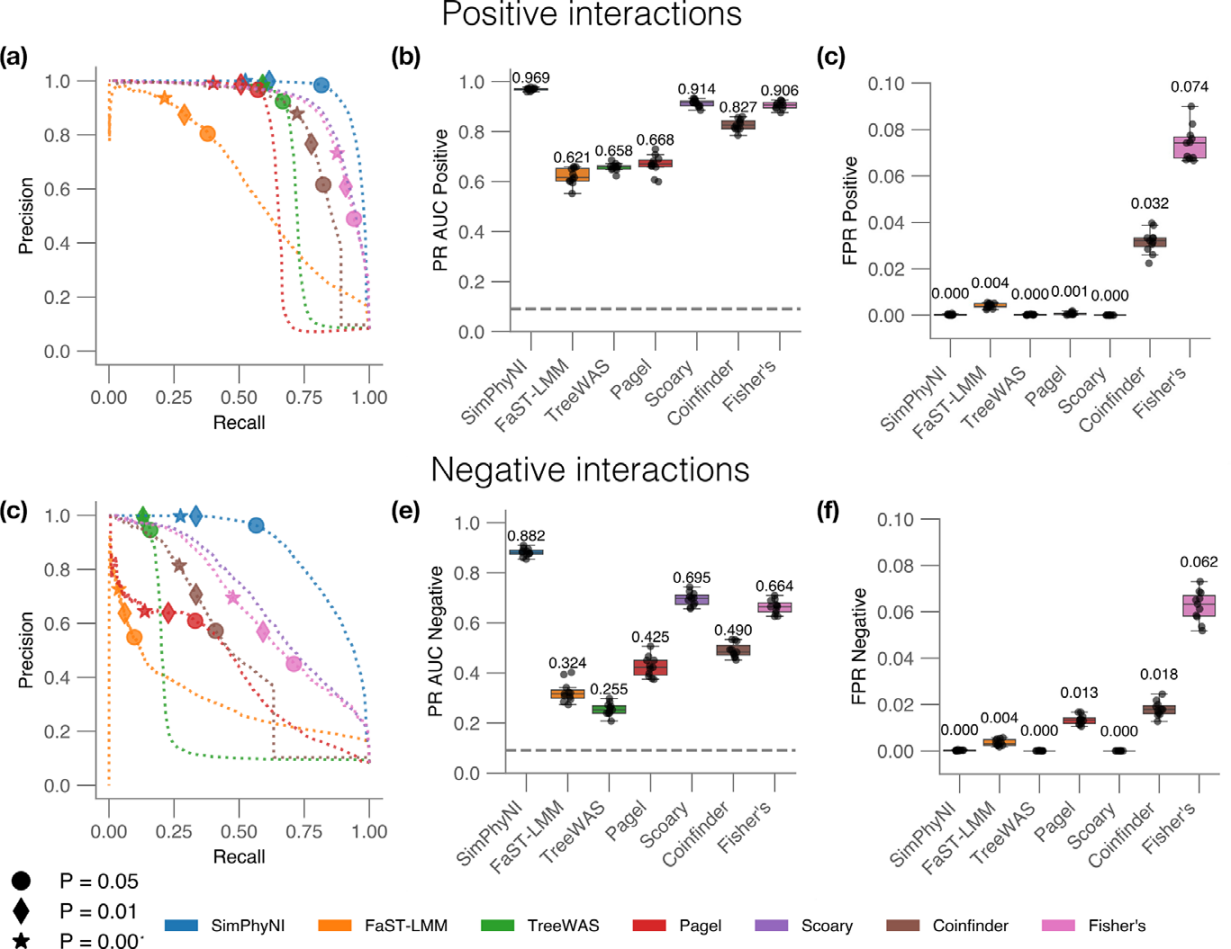
SimPhyNI shows superior performance when compared to other methods using synthetic data. We compared SimPhyNI to six other approaches (Methods) for both positive (top row) and negative (bottom row) associations. Each method is in a different colour, indicated at the bottom of the figure. (**a, d**) Precision–recall curves for tested methods. Multiple hypothesis correction adjusted *P*-value thresholds of 0.05 (circles), 0.01 (diamonds) and 0.001 (stars) are indicated for each method, except for Scoary which uses a non-*P*-value-based ranking metric. Curves are calculated from the union of 11 synthetic datasets (11 synthetic trees, 3,600 interactions each) with a 1:1:10 ratio of positive:negative:no association pairs. (**b, e**) Precision–recall area under the curve (PR AUC) values for each method using 11 synthetic datasets. Box plots show interquartile range and full range across tree typologies. Values closer to 1 indicate better performance, while values closer to 0.09 indicate near-random performance (dotted line). (**c, f**) FPRs for each method using 11 synthetic datasets. Box plots show interquartile range and full range across tree typologies. Values closer to 0 indicate better performance. Note that methods can show good performance on an individual metric but be suboptimal overall. For example, Scoary and Fisher’s both show high PR AUC, but the former has no significant associations after multiple hypothesis correction, and the latter does not achieve high precision at any threshold. See Fig. S9 for performance at different simulated interaction strengths, Fig. S10 for FPR across phylogenetic distribution metrics (Methods) and Table S3 for a statistical comparison of each metrics compared to SimPhyNI.

SimPhyNI was benchmarked against several existing methods, including TreeWAS (1.1) [8], FaST-LMM [10] implemented in pyseer (1.3.0) [3], Pagel’s correlation method as implemented in the fitPagel function in the phytools R package (2.4–4) [9], Scoary2 (0.0.25) [12], Coinfinder (1.2.1) [24] and Fisher’s exact test, a phylogenetically unaware statistical baseline for binary trait association (Fig. 3). All tools were run using the default or recommended parameters specified in their respective publications.

Performance metrics for each tool were calculated based on the average of 11 independent synthetic datasets at the tested interaction strength (a total of 11 trees and 39,600 interacting trait pairs; see section Synthetic dataset construction). Performance was evaluated at a standardized FDR *P*-value threshold of 0.01 as assessed using BY using standard classification metrics for positively and negatively associated pairs, including precision, recall and FPR. In addition to threshold-based evaluations, the threshold-independent metric precision–recall area under the curve (PR AUC) was calculated across reported *P*-value thresholds (for most methods) or rankings (Scoary).

To further evaluate performance across a broader range of population structures, we additionally benchmarked all methods on synthetic datasets generated using 11 real bacterial phylogenetic trees from the PanX database (*Bacillus subtilis*, *Bordetella pertussis*, *E. coli*, *Helicobacter pylori*, *Klebsiella pneumoniae*, *Listeria monocytogenes*, *M. tuberculosis*, *Pseudomonas aeruginosa*, *Salmonella enterica*, *Staphylococcus aureus* and *Streptococcus pyogenes*). For each tree, trait parameters were sampled from ACR of annotated pangenome gene presence/absence annotations, and synthetic interacting trait pairs were generated and evaluated as described above (Fig. S11).

### Comparative analysis with previously identified *E. coli* phage defence system associations

**Fig. 4.**
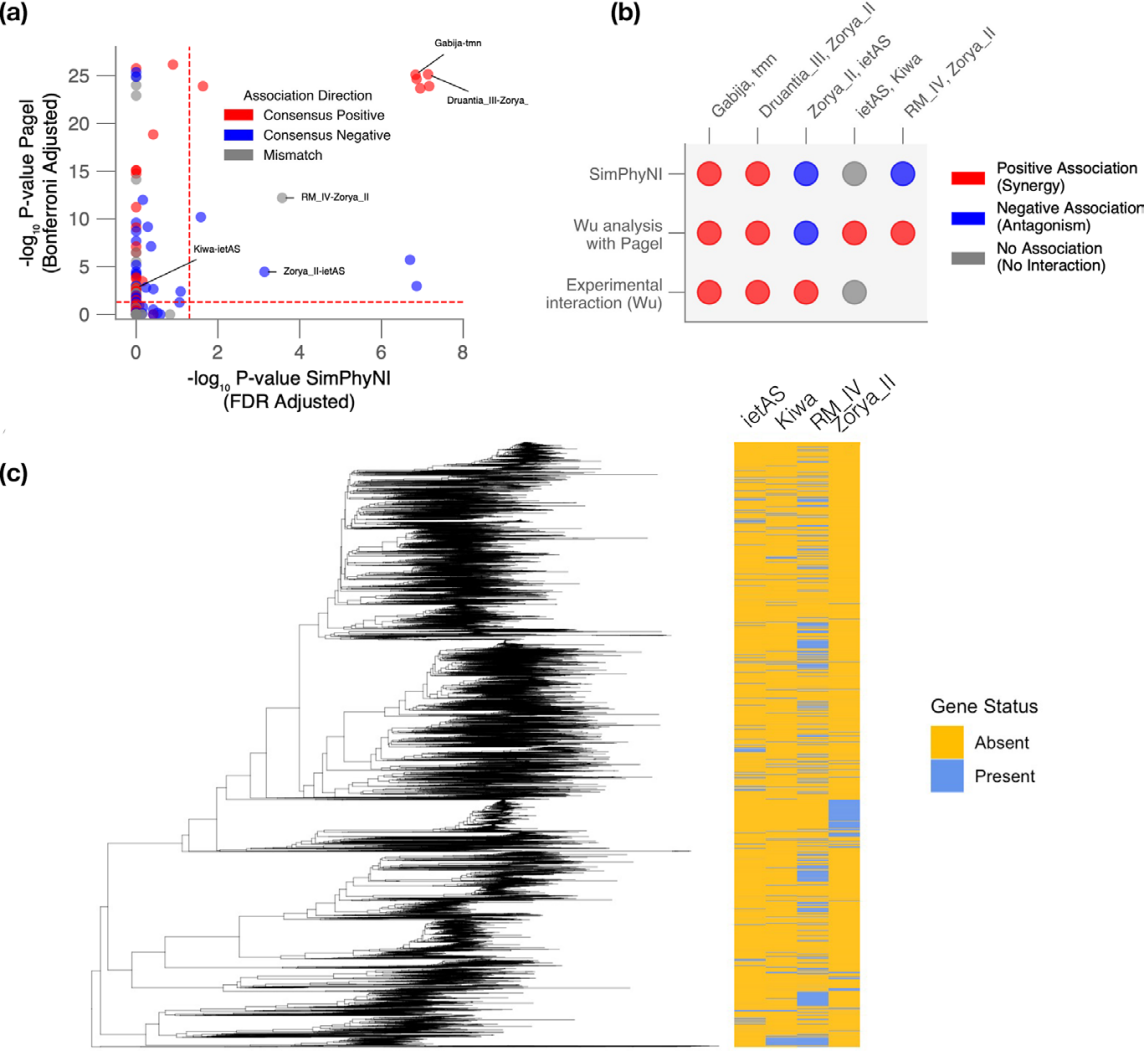
SimPhyNI corroborates known experimentally validated phage defence system associations in *E. coli*. Wu *et al*. [25] annotated phage defence systems, analysed their co-occurrence using a variant of Pagel’s correlation method and tested four pairs of systems experimentally. (**a**) Comparison of *P*-values from SimPhyNI (FDR adjusted) and Pagel’s correlation method (Bonferroni corrected) for all associations between traits with a minimum prevalence of 5% as determined by either method (Table S4). Red and blue points represent positive and negative associations, respectively, detected by both tools. Grey points indicate system pairs that had conflicting inferred interaction direction between the two methods. Dashed lines indicate significance thresholds after correction. (**b**) Results of the four experimentally tested interactions (bottom row, first four columns) compared to predictions from SimPhyNI (top row) and Pagel’s method (middle row). Red indicates a predicted or validated positive interaction, blue indicates a negative interaction, and grey indicates no interaction. One additional gene pair (RM type II and Zorya II) with conflicting directionality despite strong significance in both tools is shown. (**c**) The distribution of pairs of systems with conflicted predictions is shown in more detail, with the *E. coli* phylogenetic tree from Wu *et al*. [25] on the left and presence and absence of systems indicated on the right.

We re-analysed a dataset comprising 26,362 *E. coli* genomes previously examined by Wu *et al*. [25] (Fig. 4). This dataset included a neighbour-joining (NJ) phylogenetic tree and reported annotations of phage defence systems. In their study, Wu *et al*. employed Pagel’s correlation method with uniform branch lengths (in contrast to the traditional Pagel’s test benchmarked here) to identify significant co-occurrence and exclusion patterns among defence systems, applying a stringent Bonferroni correction (*α*=0.05) for multiple testing. The same phylogenetic tree and defence system annotations were input into the SimPhyNI pipeline to compare results across methods. To better align with the statistical conventions for SimPhyNI set by this study, a BY FDR correction was applied at a threshold of 0.05. See Table S4 for full result comparison.

### Analysis of the *E. coli* accessory genome

**Fig. 5.**
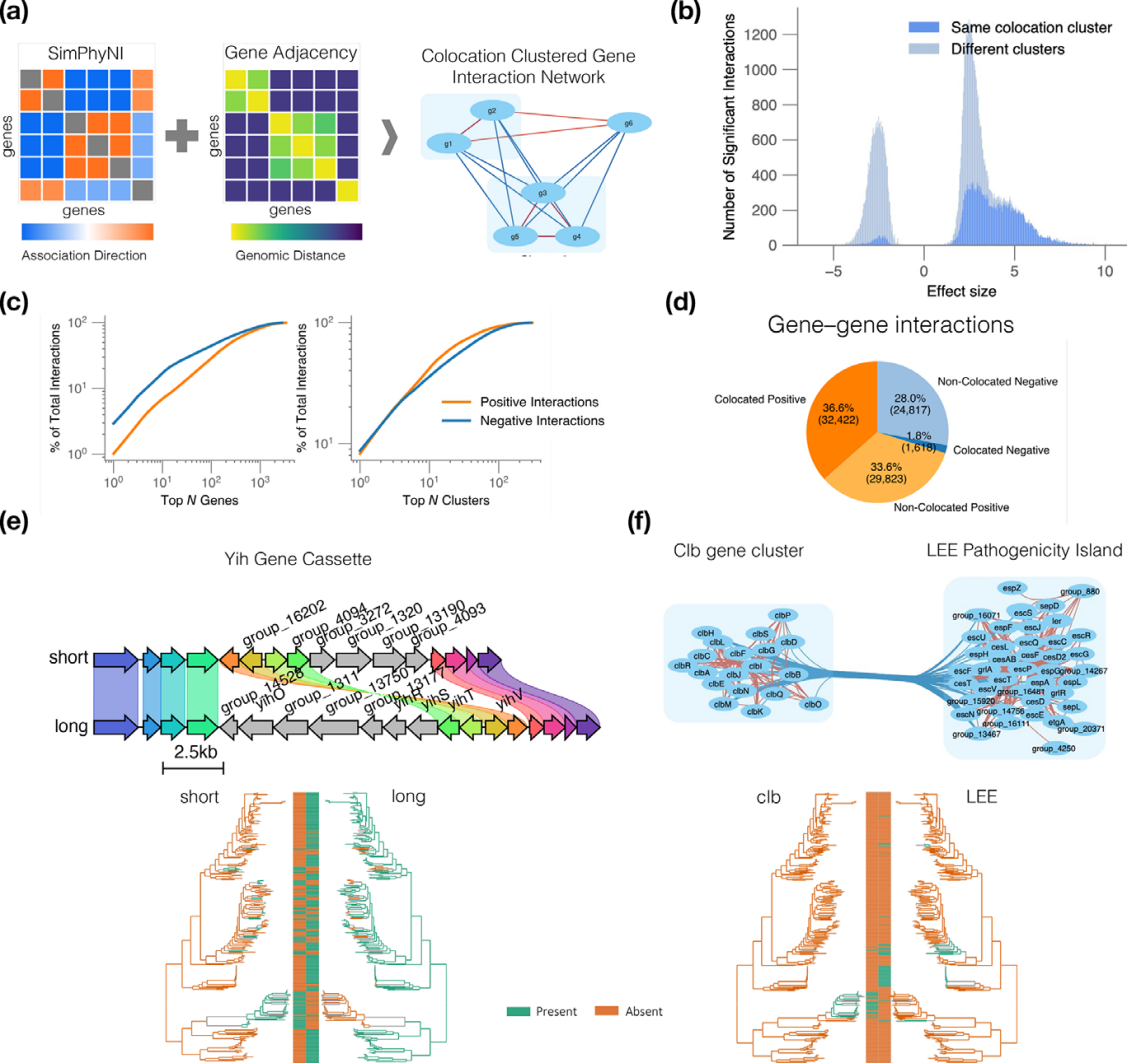
SimPhyNI identifies evolutionary interactions between gene clusters in the *E. coli* pangenome. (**a**) Gene pairs identified by SimPhyNI (left) that are identified to have shared genomic contexts (middle) are grouped together and assigned to the same colocation cluster (right). (**b**) Stacked histograms of effect sizes for statistically significant interactions (BY<0.01). Gene pairs within the same colocation are indicated in dark blue. Positive effect sizes indicate inferred positive associations; negative effect sizes indicate inferred negative associations; only statistically significant interactions are shown. (**c**) Cumulative percentage of colocation-filtered interactions among top-ranked genes and gene clusters. Genes and clusters are ranked by the number of significant interactions in which they participate (high to low), illustrating that some genes and clusters contribute disproportionately to the interaction network. (**d**) Pie chart indicating the proportion of significant positive and negative gene–gene interactions within and between colocation clusters. (**e, f**) Two examples of negatively interacting clusters. (**e**) Two structural variants of the *yih* gene cassette were identified as negatively interacting; an alignment showing homologous flanking regions for both variants, named here ‘long’ and ‘short’ cassettes. Genes unique to each cassette version comprise the clusters and are labelled on the sequence alignment. The phylogenetic tree and heatmap (bottom) show co-occurrence patterns across the *E. coli population*. This negative interaction has been recently described [26]. (**f**) Interaction network showing negative associations between the *clb* gene cluster and the LEE pathogenicity island, both associated with *E. coli* virulence, alongside phylogenetic co-occurrence patterns across the *E. coli* population. See Table S5 for all significant interactions.

A representative collection of 500 *E. coli* genomes chosen by PanX [1] and the corresponding maximum likelihood phylogenetic tree were downloaded from the NCBI and PanX, respectively. Genomes were annotated using Prokka (1.14.6) [26], and genes were clustered with Panaroo (1.5.2) [2] using the built-in moderate cleaning mode with removal of invalid genes and merging of paralogues. The pangenome output was then filtered for genes between 5 and 95% prevalence. The resulting 4,333 accessory genes were tested for associations using SimPhyNI. The resulting 9.4 million tests were corrected using a 0.01 BY FDR adjustment (Fig. 5, Table S5).

To remove associations confounded by colocation, a gene graph was built using average genomic distance as edges. Edges were drawn if genes were within 50 kb (the scale of a large plasmid). The graph was then clustered using the Leiden graph neighbourhood algorithm into 359 gene clusters at a resolution of 2. Varying the resolution from 1 to 2 resulted in only a modest increase in cluster number (326 to 359), indicating that the inferred gene groupings were robust to this parameter choice. Interactions between clusters were aggregated and evaluated for consistency, mean effect size and density (reported in Table S5). In order to further filter significant interactions, phylogenetic spread for each gene was calculated as the mean of pairwise distances between all genomes in which the gene was present normalized by the maximum possible pairwise distance on the phylogenetic tree (Fig. S15).

When evaluating specific cluster–cluster interactions, we assessed negatively associated clusters for conserved genomic context using Clinker (v0.0.31) [27], performing local alignments of complete gene clusters with ten upstream and ten downstream flanking genes to identify shared genomic contexts that could explain mutual exclusivity.

### Analysis of *M. tuberculosis* rifampicin resistance mutations

SimPhyNI, FaST-LMM and Fisher’s exact test were applied to whole-genome sequencing data and corresponding resistance data from the CRyPTIC Consortium (v2.0) [28]. These three methods were selected on the basis of computational efficiency, scalability to large cohorts and capacity to accept genome-wide feature sets. All methods were used to find associations between genome-wide SNVs and indels and the rifampicin (RIF) resistance phenotype across 59,353 *M*. *tuberculosis* isolates (30.6% resistant). Binary phenotypes were derived from drug susceptibility test measurements by retaining only susceptible (S) and resistant (R) calls, using only measurements with higher quality scores for samples with replicates and excluding irreconcilable S/R conflicts. A variant feature matrix was constructed from consensus-level mutation calls with read coverage ≥5, retaining mutations observed in at least 5 samples (195,282 variants). Ground truth was provided by the World Health Organization (WHO) resistance mutation catalogue [29], yielding 47 causal resistance mutations after filtering. For each method, BY correction was applied at *P*<0.01 over the shared variant set, and performance was assessed by recovery and ranking of WHO-R variants (Fig. S16). Full results are available in Table S6.

## Results

### Development and validation of SimPhyNI using synthetic data

SimPhyNI, like previous microbial trait association methods, is centred around null models of trait–gene and gene–gene co-occurrence derived via simulation [8, 12, 13, 30].

We found that downstream evaluation metrics were sensitive to implementation choices for the trait evaluation Markov Model (native vs uniform branch length, including or excluding an emergence threshold, continuous vs discrete time, etc.) (Table S1, Fig. S1). Similarly, we observed that the choice of scoring function substantially influenced both statistical power and FPRs (Methods; Table S2, Fig. S1). Therefore, we devised an approach to optimize both of these critical steps. To systematically identify the optimal combination of simulation method and scoring function, we compared performance across sets of approaches using synthetic data (Methods). All method combinations were evaluated using BY FDR correction at a threshold of 0.01.

To perform well in these synthetic datasets, a method must (1) maintain high statistical separation of neutral traits associations versus positive or negative associations, (2) display consistent performance across variable tree structures and (3) recover interactions even after correction for multiple tests. The best-performing configuration combined a log-odds scoring function with a branch length-aware Markov simulation (Methods). This simulation process uses asymmetric gain and loss rates learnt from the ACRs of traits, then initializes the root of each simulation with the inferred root state ACR and applies a novel parameter, emergence threshold, before which no state changes are simulated (Methods). The combination of simulation method and this scoring function yields exceptional PR AUC values, 0.969 for positive and 0.882 for negative associations, and the low FPRs (0.0002 and 0.0002) after correction (Fig. S1). Given a baseline PR AUC for a random classifier near 0.09 and FPR ~0.9 for this imbalanced classification task, these results reflect strong discriminatory performance. We also note that continuous time simulation also performed well with all metrics being statistically similar to our discrete time method (Fig. S1). We chose the latter for its computational efficiency and simplicity.

### SimPhyNI outperforms existing methodologies for trait associations in synthetic data

SimPhyNI was benchmarked against five established association-detection methods developed for microbial traits: Pagel’s correlation [9], FaST-LMM [10], TreeWAS [8], Scoary [12, 13] and Coinfinder [24]. Fisher’s exact test, a basic test for assessing associations between two binary variables, was also included as a baseline method that is not corrected for phylogeny.

Pagel’s correlation is a rigorous likelihood-based framework that compares models of dependent and independent trait evolution on a given phylogenetic tree [9] and only works on binary traits. FaST-LMM [10] and TreeWAS [8] are newer methods designed for broader trait applications, including continuous and multi-state discrete traits. FaST-LMM employs a linear mixed model and a kinship matrix to capture the covariance structure among samples [11]. TreeWAS, like SimPhyNI, is simulation-based; however, it differs in implementation details and, more significantly, in the evaluation of trait co-occurrence. TreeWAS natively contains three separate scores for evaluation of interactions; we observed similar trends in evaluation metrics across all three scores, and thus, only the terminal score is shown in Fig. 3 (full results in Fig. S9). Scoary [12, 13] is a tool for efficient gene presence or absence association testing across a bacterial pangenome. Scoary applies label-switching permutation to filter putative results from Fisher’s exact test. Notably, Scoary uses two distinct metrics for reporting results: a ranking metric (Fisher’s *q*-value multiplied by the permutation *P*-value) and a validation metric (permutation *P*-value alone); we used the ranking metric for PR AUC calculations and the validation metric for FPR. Coinfinder [24] is designed specifically for binary presence/absence data and outputs a network of significantly associated trait pairs. The same synthetic datasets generated above for optimization were used here for benchmarking across these tools.

Precision–recall analysis demonstrated SimPhyNI’s increased performance compared to these tested methods, achieving average AUC scores of 0.969 and 0.882 for positive and negative associations, respectively, which is higher than all alternative approaches (Mann–Whitney U test, *P*<10^−4^) (Fig. 3a, b, d, e, Table S3). All tested methods consistently outperformed the baseline AUC of 0.09 expected from the class imbalance of the test set. TreeWAS, FaST-LMM and Pagel’s correlation had lower PR AUC values compared to other methods (<0.70 for positive and <0.50 for negative interactions) due to their lower recall – likely reflecting their design to prioritize high positive predictive value. A high PR AUC for Scoary (0.914 and 0.695) indicates high separation between interacting and non-interacting traits, performing second to SimPhyNI in both positive and negative associations, and highlights the quality of its ranking design [13] (Fig. 3b, e). Coinfinder shows middle-of-the-pack separation of hypotheses with PR AUCs of 0.827 and 0.490. Fisher’s exact test surprisingly showed high separation in this dataset for positive interactions (AUC=0.906); however, at the standard *P*-value threshold of 0.01, Fisher’s had a very high FPR (Fig. 3c, f), and Fisher’s performed poorly when traits show high lineage-based structure with few evolutionary events or high prevalence (Fig. S10).

A key strength of SimPhyNI lies in its ability to minimize false positives. Most gene pairs are expected to show no interaction, making stringent FPR control essential [8]. SimPhyNI exhibited consistently near-zero FPRs (0.0002) for both positive and negative associations (Fig. 3c, f). Multiple other tested phylogenetic methods have similarly low FPRs. Scoary – using the permutation test *P*-value – did not have any tests that were significant after multiple hypothesis correction in any dataset. Though not scalable using standard statistical conventions, we do acknowledge the ability of Scoary to rank hypotheses for potential interaction. Aside from Scoary, the only case that was similar in FPR to SimPhyNI was TreeWAS with statistically similar FPR for positive associations and slightly lower FPR for negative associations (0.0002 vs 3e-5, Table S3). This improved negative FPR comes at the cost of reduced recall for TreeWAS. All other methods have significantly higher FPR than SimPhyNI. Coinfinder and Fisher’s exact test have the highest FPRs for both positive and negative associations. Paired with our analysis of PR AUC, it is clear that SimPhyNI achieved higher statistical separation, retaining low FPRs while recovering more interactions compared to other methods.

All methods detected positive interactions better than negative interactions (Fig. 3). This difference likely arises from inherent asymmetries in detecting trait interactions. Negative associations are harder to distinguish from null models than positive associations, especially under low trait prevalence conditions; for example, two traits at 10% prevalence are not expected to overlap much even when non-interacting. Additonally, negative interactions between traits can lower their prevalences, further complicating detection. SimPhyNI’s log-odds scoring method helps to overcome this problem by implicitly incorporating the prevalence of each trait in its null model (Methods). This adjustment enables robust performance when trait frequencies vary (Figs S1 and S10). As a result, SimPhyNI can distinguish genuinely negative interactions from neutral cases.

We repeated benchmarking across a range of interaction strengths and phylogenetic parameters and found that SimPhyNI consistently outperformed all other tools at separating interacting and non-interacting traits (Figs S9 and S10). To confirm that these results generalize across a broader range of population structures, we additionally benchmarked all methods on synthetic data generated using 11 real bacterial phylogenetic trees from the PanX database [1], with trait parameters sampled from empirical ACR of annotated pangenome genes. SimPhyNI maintained superior PR AUC and near-zero FPR across all species trees tested, consistent with our primary benchmarking results (Fig. S11).

Lastly, we compared computational efficiency using 27 synthetic datasets across 3 different tree sizes (100, 500 or 1000 leaves) and 3 different trait counts (10, 100 or 1000 traits), analysing all pairwise interactions in each tree. SimPhyNI takes time to compile key operations to machine code, resulting in sizeable overhead that causes marginally slower but manageable run times on small datasets when compared to other efficient tools (100 leaves and 10 traits, Fig. S12). On larger datasets (>100 leaves and >10 traits), we observed SimPhyNI to be faster than all other tested methods (Fig. S12), demonstrating that SimPhyNI’s high precision and recall do not come at the cost of computational efficiency.

### SimPhyNI accurately recovers biologically supported interactions among phage defence systems in *E. coli*

We next sought to demonstrate the utility of SimPhyNI in real-world biological datasets. Prior studies have hypothesized that the evolutionary associations between phage defence systems may be predictive of synergistic defence against phage [25]. To further demonstrate the use of SimPhyNI, we reanalysed the 26,362-genome *E. coli* dataset from Wu *et al*. [25], which computationally inferred synergistic anti-phage activity in *E. coli* and validated a subset of putative interactions between defence systems. The original study employed Pagel’s correlation method with uniform branch lengths (uniform Pagel) to identify co-occurrence and exclusion patterns among phage defence systems. We processed the same NJ phylogeny and phage defence annotations through the SimPhyNI pipeline.

Across the 2,485 trait pairs tested by both methods, we observed substantial divergence in the significant associations identified (Fig. 4a). Only 11 associations were deemed significant by both SimPhyNI and uniform Pagel. Uniform Pagel exclusively identified 51 pairs, while all interactions detected in SimPhyNI were detected with uniform Pagel – though one had different predicted interactions between the tests. This is consistent with our synthetic benchmarking, where Pagel’s demonstrates higher FPRs (Fig. 3). The use of uniform branch lengths in the Wu *et al*. study likely further contributes to this gap with uniform values removing approximate evolutionary time information that would be used to evaluate evolution models in the test.

Wu *et al*. experimentally tested four computationally predicted defence system interactions. Two predicted interactions aligned with experimental outcomes: positive associations between Gabija and Tmn as well as Druantia III and Zorya II both showed mechanistic synergy in antiphage activity. However, a predicted negative association between Zorya II and IetAS was also shown to exhibit mechanistic synergy in antiphage activity experiments (Fig. 4b). Wu *et al*. suggested that this conflicting case may reflect other evolutionary drivers for the separation of these systems such as metabolic costs or functional redundancy. The fourth prediction, a positive association between IetAS and Kiwa, was found to have no detectable non-additive antiphage activity.

When we applied SimPhyNI to these same interactions, our computational predictions showed stronger concordance with Wu *et al*.’s experimental findings (Fig. 4b). SimPhyNI predicted no interaction between IetAS and Kiwa, which corroborates experimental findings. Further, among the 12 shared significant associations between the two methods, we found one case of directional conflict: the association between RM type IV and Zorya II was called positive by Pagel’s method but negative by SimPhyNI. Visual inspection of the trait distributions on the phylogenetic tree revealed localized patterns of mutual exclusion (Fig. 4c), suggesting SimPhyNI’s inference of a negative relationship to be more fitting. These results suggest that SimPhyNI represents a viable and potentially superior alternative approach for large-scale trait associations in phylogenetic contexts.

### SimPhyNI enables high-throughput pangenome analysis, identifying biologically meaningful interactions

The power and scalability of SimPhyNI enable analyses that might be computationally prohibitive with existing methods. To demonstrate this capability, we performed a pangenome-wide association screen testing all 9.4 million possible pairs of 4,333 accessory genes across 500 *E. coli* genomes. This exhaustive pairwise analysis was completed in ~10 min when parallelized (64 cores, 64 gb RAM total). SimPhyNI identified 62,245 positive interactions and 26,435 negative associations after multiple-hypothesis correction (Methods).

Physical genomic proximity is a key competing hypothesis for co-occurrence associations. Genes coinherited on the same mobile element or syntenic cluster can show strong associations driven by shared genomic context rather than epistatic interaction. To distinguish these cases, we constructed a colocation graph from pairwise genomic distances between significantly associated gene pairs and used Leiden clustering to identify communities of co-located genes (Methods, Figs 5a and S13). This resulted in 32,422 positive interactions contained within colocation clusters and, interestingly, 1,618 negative interactions contained within colocation clusters (Fig. 5b, d). These negative interactions highlight non-homologous genes that have shared genetic neighbourhoods, yet rarely exist together in the same genome, likely reflecting mobile elements that compete for integration sites or functional submodules.

When analysing trends across the *E. coli* pangenome, we observed non-uniform contributions from genes and gene clusters, indicating hubs for evolutionary interactions (Fig. 5c). This finding aligns with prior studies that also show hub-like organization of gene–gene associations in the *E. coli* pangenome [31]. However, we noticed some likely false-positive interactions resulting from inferred gains and losses within a poorly resolved ‘outbreak-like’ cluster of the phylogeny, resulting in inaccurate trait simulations (Fig. S14). We therefore used a metric for phylogenetic spread (Methods; Fig. S15) to sort through interactions alongside effect size. To highlight the increased performance of SimPhyNI on negative associations, we focus on notable examples of predicted antagonism below.

The largest negative interaction between two colocation clusters was also phylogenetically dispersed (mean effect size of −4.71). These clusters contain two distinct structural variants of the *yih* gene cassette (Fig. 5e). The *yih* gene cassette is responsible for the catabolism of sulfoquinovose, a plant-derived sugar used as a sulphur and carbon source and is present in >95% of *E. coli* within our pangenome study. The structural variants (long and short cassette) differ by a four-gene inversion and unique genetic content; their scattered distribution across the phylogenetic tree suggests frequent recombination (Fig. 5e). Their structural and metabolic differences were recently characterized [32], confirming the biological significance of this interaction. While previous methods could likely detect the mutual exclusivity of these variants, SimPhyNI’s scalability and prioritization of results through effect size ranking make this biologically meaningful association immediately evident.

SimPhyNI also highlighted interactions that may reflect novel ecological or pathogenic trade-offs in *E. coli*. We identified a strong negative association between the colibactin (clb) genotoxin island and the LEE pathogenicity island (Fig. 5f). These two loci both promote gut colonization of pathogenic *E. coli* and do not share a gene neighbourhood (Methods). The clb cluster produces colibactin, a genotoxin that slows epithelial turnover and dampens immune responses [33], whereas the LEE island encodes a type III secretion system that remodels the host epithelium and promotes adherence [34]. Though both confer host-associated advantages, the two islands show within phylogroup mutual exclusivity with an average effect size of −2.37 (Methods). This pattern suggests that maintaining both virulence systems simultaneously is either metabolically costly, functionally redundant or ecologically disadvantageous – all hypotheses that could be evaluated through future experimental work.

Together, these results illustrate how SimPhyNI can be used to effectively analyse and order large-scale genome-wide data, recover known interactions and provide hypotheses for further mechanistic study within microbes. A list of all putatively interacting gene and cluster pairs in *E. coli* is reported in Table S5 to serve as a resource for the community

### SimPhyNI recovers known causal variants for rifampicin resistance in *M. tuberculosis* at large scale

To further assess SimPhyNI’s scalability and utility beyond gene presence/absence data, we applied it alongside FaST-LMM and Fisher’s exact test to genome-wide variant associations in *M. tuberculosis*. These three methods were selected on the basis of computational efficiency on this large feature set. We tested 195,282 variants for associations with rifampicin (RIF) resistance across 59,353 isolates from the CRyPTIC Consortium (Methods).

To compare performance across methods, we looked at the ranking of 47 known causal resistance mutations catalogued by the WHO mutations [29]. The performance of all 3 methods correlated with variant frequency, with low-frequency variants (29/47 variants with <50 observations) remaining undetectable regardless of method (Fig. S16). Among the detectable variants, SimPhyNI recovered more WHO-catalogued causal mutations than FaST-LMM (11/47 variants compared to 3/47), with comparable rankings for the hits shared between the two methods (top three causal variants in top five hits). While Fisher’s exact test had higher recall than SimPhyNI (18/47 variants), it also had significantly higher total hits (988 vs 10,695 total hits), consistent with its high FPR (Fig. 3c). A number of significantly associated variants were identified at high rankings from SimPhyNI and FaST-LMM, though not designated as causal of RIF resistance by WHO (Table S6). Many of these hits reflect known compensatory mutations in genes such as *rpoC* and *rpoA* that restore fitness in resistant strains [35], as well as co-carried resistance mutations common in multi-drug-resistant lineages [36, 37], highlighting the need for context-dependent biological interpretation for any association study. Together, these findings demonstrate that SimPhyNI’s phylogenetically informed null model scales effectively to large cohorts and SNV-level feature spaces.

## Discussion

We have shown that SimPhyNI achieves high precision and recall in identifying evolutionary interactions (Fig. 3). Notably, it (1) excels in detecting negative associations (antagonisms), which are frequently missed by other tools, and (2) achieves near-zero FPRs, which is critical due to the sparsity of true interactions relative to all possible trait pairs. This robustness makes SimPhyNI reliable for exploratory analyses where spurious hits could otherwise overwhelm interpretation. We show multiple use cases of SimPhyNI’s accuracy, including targeted searches on large phylogenetic trees (Fig. 4), network recovery with large numbers of gene–gene interactions (Fig. 5) and standard genotype–phenotype mGWAS with large numbers of variants (Fig. S16).

SimPhyNI’s strong statistical performance was enabled by two key innovations. First, its null simulation of independent traits generates a stable distribution that accurately reflects input data (Fig. S5); key features include a novel method for transition rate calculation (Fig. S6) and a time-to-first-event parameter that prevents simulated emergence on deep branches where empirical trait ancestral states are too poorly resolved to support reliable transition inferences (Fig. S5, Methods). Second, we score co-occurrence using a log-odds function that incorporates all four joint states of a tested trait pair. This function both accounts for variation in trait prevalence across simulations and enables the detection of negative associations (Figs 3 and S1, Table S2).

Moreover, SimPhyNI achieves superior efficiency (Fig. S12) using two key innovations. First, stable *P*-values for co-occurrence comparisons are generated from a small number of simulations (generally 64) using a KDE to approximate null co-occurrence distributions from the combinatorial comparison of simulated traits (Fig. S2). Second, though implemented in python, we compile key functions for ACR and null model generation to machine code for space and time efficiency. These improvements drastically reduce computational cost, thus enabling large-scale analyses of genome-wide epistasis and mGWAS (Figs 5 and S16).

Although SimPhyNI achieves high precision while maintaining high recall, detecting interactions in evolutionary data remains inherently challenging for at least two reasons. First, interacting trait pairs with low prevalence or limited phylogenetic spread (phylogenetic *D*<0; Methods) may remain undetectable. While we cannot overcome this limitation, we show SimPhyNI maintains near zero FPRs across at low prevalence and spreads (Fig. S10). Second, trait co-occurrence can be driven by signals beyond causation or epistasis – such as physical colocation on the genome or shared environmental pressures. Post-SimPhyNI steps can be implemented to detect or reduce the impact of such non-causal correlations. For example, we demonstrate how genomic distance-based clustering can reduce non-causal correlations (Figs 5 and S13). Users of SimPhyNI are encouraged to consider incorporating covariates such as host, sampling site or environment as binary features within SimPhyNI for post hoc filtering.

While SimPhyNI is currently restricted to binary traits, the underlying simulation framework is readily extendable to multi-state and continuous traits through larger Markov transition matrices or Brownian motion models, and the current log-odds scoring function can be generalized to multiclass likelihood statistics. These extensions could broaden the scope of SimPhyNI to encompass useful metrics like expression levels, growth rate and minimum inhibitory concentrations. Thus, while SimPhyNI shows excellent performance for binary traits, which are central to many questions in microbial evolution (including gene presence/absence, binary phenotypes and disease incidence), its flexible design positions it as a foundation for future models that accommodate a broader range of microbial trait evolution.

Altogether, SimPhyNI is a robust, extensible framework for evolutionary interaction inference, with computational efficiency that opens new avenues for large-scale exploration of associations and co-evolution in microbial genomes.

## Supplementary material

10.1099/mgen.0.001791 Supplementary Material 1.

10.1099/mgen.0.001791 Supplementary Material 2.

## Abbreviations

ACR: ancestral character reconstruction
BH: Benjamini–Hochberg
BY: Benjamini–Yekutieli
FDR: false discovery rate
FPR: false positive rate
GWAS: genome-wide association study
HGT: horizontal gene transfer
KDE: kernel density estimation
mGWAS: microbial genome-wide association study
MPPA: marginal posterior probability approximation
NJ: neighbour joining
PR AUC: precision–recall area under the curve
SimPhyNI: Simulation-based Phylogenetic iNteraction Inference
WHO: World Health Organization
