# Supplementary material for: High-precision binary trait association on phylogenetic trees
Source: Microb Genom. 2026 Jul 31;12(7):001791. doi: 10.1099/mgen.0.001791 (PMC13427033; doi:10.1099/mgen.0.001791)
Supplement: Supplementary Material 1. [file mgen-12-01791-s001.pdf]

Supplementary Information for:

High Precision Binary Trait Association on Phylogenetic Trees

Ishaq O Balogun[1,2], Christopher P Mancuso[1,2], Tami D Lieberman[1,2]

[1]Institute for Medical Engineering and Sciences, Massachusetts Institute of Technology; Cambridge, MA 02142, USA.

[2] Department of Civil and Environmental Engineering, Massachusetts Institute of Technology; Cambridge, MA 02142, USA

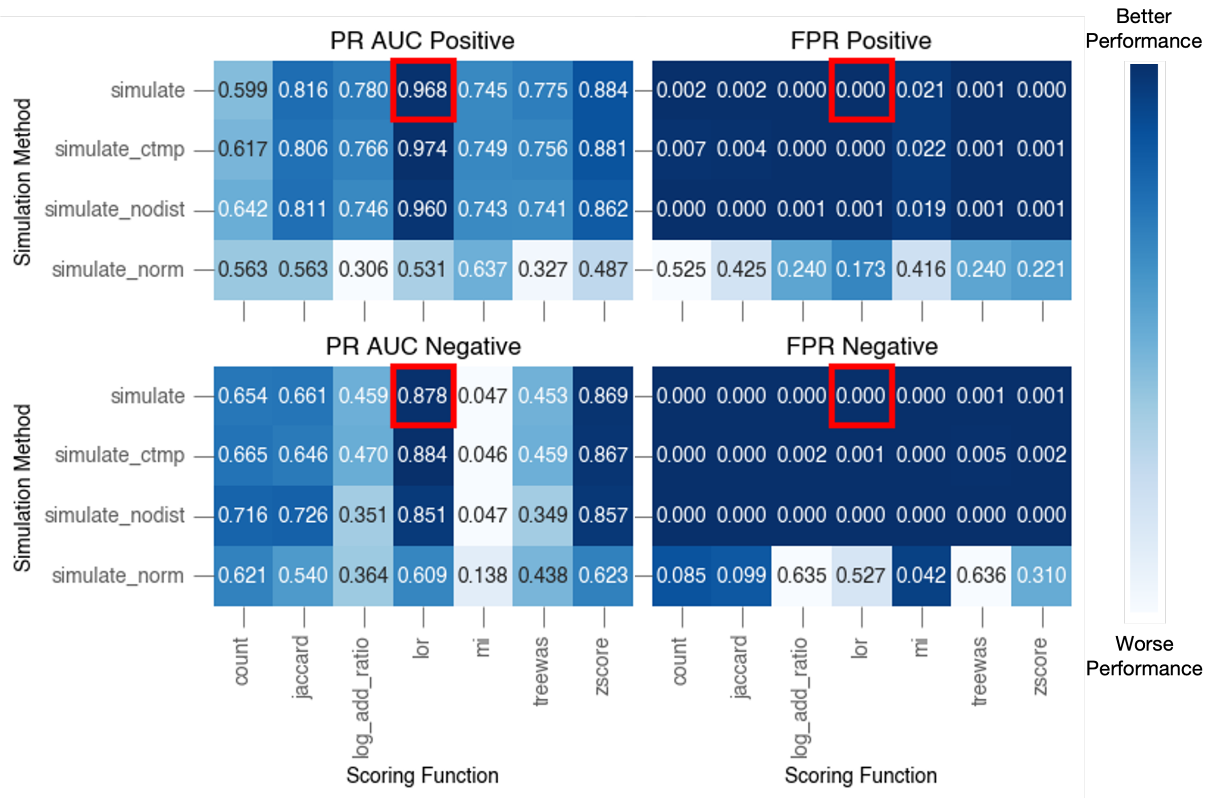

PR AUC Positive	count	jaccard	log_add_ratio	lor	mi	treewas	zscore
simulate	0.599	0.816	0.780	0.968	0.745	0.775	0.884
simulate_ctmp	0.617	0.806	0.766	0.974	0.749	0.756	0.881
simulate_nodist	0.642	0.811	0.746	0.960	0.743	0.741	0.862
simulate_norm	0.563	0.563	0.306	0.531	0.637	0.327	0.487

FPR Positive	count	jaccard	log_add_ratio	lor	mi	treewas	zscore
simulate	0.002	0.002	0.000	0.000	0.021	0.001	0.000
simulate_ctmp	0.007	0.004	0.000	0.000	0.022	0.001	0.001
simulate_nodist	0.000	0.000	0.001	0.001	0.019	0.001	0.001
simulate_norm	0.525	0.425	0.240	0.173	0.416	0.240	0.221

PR AUC Negative	count	jaccard	log_add_ratio	lor	mi	treewas	zscore
simulate	0.654	0.661	0.459	0.878	0.047	0.453	0.869
simulate_ctmp	0.665	0.646	0.470	0.884	0.046	0.459	0.867
simulate_nodist	0.716	0.726	0.351	0.851	0.047	0.349	0.857
simulate_norm	0.621	0.540	0.364	0.609	0.138	0.438	0.623

FPR Negative	count	jaccard	log_add_ratio	lor	mi	treewas	zscore
simulate	0.000	0.000	0.000	0.000	0.000	0.001	0.001
simulate_ctmp	0.000	0.000	0.002	0.001	0.000	0.005	0.002
simulate_nodist	0.000	0.000	0.000	0.000	0.000	0.000	0.000
simulate_norm	0.085	0.099	0.635	0.527	0.042	0.636	0.310

Figure S1: **Metrics for all test statistics and simulation methods using 4-state Markov data generation using Benjamini-Yekutieli correction of 0.01.** Each pair of heatmaps reports a performance metric (PR AUC or FPR) across combinations of simulation methods (rows) and scoring function (columns). The top and bottom rows report performance for the identification positive and negative associations, respectively. The log odds ratio scoring function was observed to have the highest performance across metrics. Notably our default simulation method and continuous time markov process (CTMP) variant have the best performance, with CTMP having a slight edge in in some metrics, however the difference was not statistically significant, making us favor the more computationally efficient default method. See Table S1 for definitions of the tested simulation methods and Table S2 for definitions of the tested scoring functions.

Table S1: Simulation Methods Implemented in SimPhyNI

**Simulation Method**	**Implementation Details**
`base simulation`	Markov process with a maximum of one event per branch. Uses a distance threshold, before which no events are simulated.
`ctmp`	Continuous-time implementation of `base simulation` allowing multiple events per branch using exponential waiting times.
`nodist`	`base simulation` implementation without a distance threshold for trait emergence.
`norm`	`base simulation` with all branch lengths normalized to a fixed value (1).

Table S2: Scoring Functions for Trait Co-occurrence

Scoring Function	Formula	Observations
Jaccard Index	$\frac{A \cap B}{A \cup B}$	Simple similarity metric. Easy to interpret but sensitive to low prevalence. Limited statistical power.
Log add ratio	$\log\left(\frac{(A \cap B) + (\neg A \cap \neg B)}{(\neg A \cap B) + (A \cap \neg B)}\right)$	Emphasizes co-occurrence and co-absence over mismatches. Sensitive to imbalanced marginals.
Log odds ratio (LOR)	$\log\left(\frac{(A \cap B)(\neg A \cap \neg B)}{(A \cap \neg B)(\neg A \cap B)}\right)$	Statistically powerful. Used in SimPhyNI null model testing. Performs well across prevalence spectra.
Mutual Information (MI)	$\sum_{A,B \in \{0,1\}} P(A \cap B) \log\left(\frac{P(A \cap B)}{P(A)P(B)}\right)$	Measures dependency between presence/absence vectors A and B. Symmetric. Sensitive to sampling noise. Requires smoothing with small sample sizes.
TreeWAS Terminal Statistic (treewas)	$(A \cap B) + (\neg A \cap \neg B) - (\neg A \cap B) - (A \cap \neg B)$	Captures agreement between predicted and observed trait values across descendant nodes. Used in TreeWAS to evaluate phylogenetically corrected association strength.
Count	$A \cap B$	Simple co-occurrence count. Does not adjust for background rates. Biased by prevalence.
Z score	$\frac{A \cap B - P(A)P(B)N}{P(A)P(B)(1 - P(A)P(B))}$	Standardized deviation from expected co-occurrence. Can inflate importance with large N.

Legend:
N : Total number of genomes or samples.
A, B : Binary vectors indicating trait presence/absence across N samples.
$A \cap B$: Count of samples where both traits are present.
$A \cup B$: Count of samples where either trait is present.
$\neg A$: Complement of A (absence of trait A).
$P(A)$, $P(B)$: Proportions of samples with traits A, B respectively.
ϵ (not shown): Small positive pseudocount used for smoothing (typically 1).

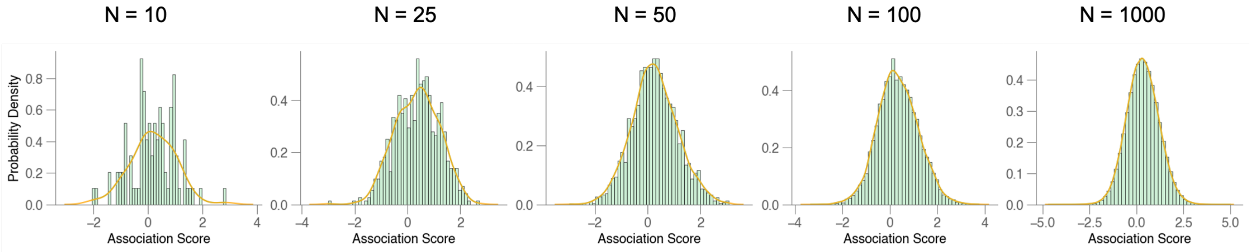

Figure S2: **Comparison of KDE versus discrete null distributions for trait co-occurrence scores.** Presented as as visual for null co-occurrence distribution approximation using kernel density estimation. N represents the number of simulations used for each trait therefore plots show N^2 co-occurrence observations corresponding to the exhaustive comparison of trait simulations. As N increases the discrete observations (green bars) and KDE (orange line) converge to the same normal distribution.

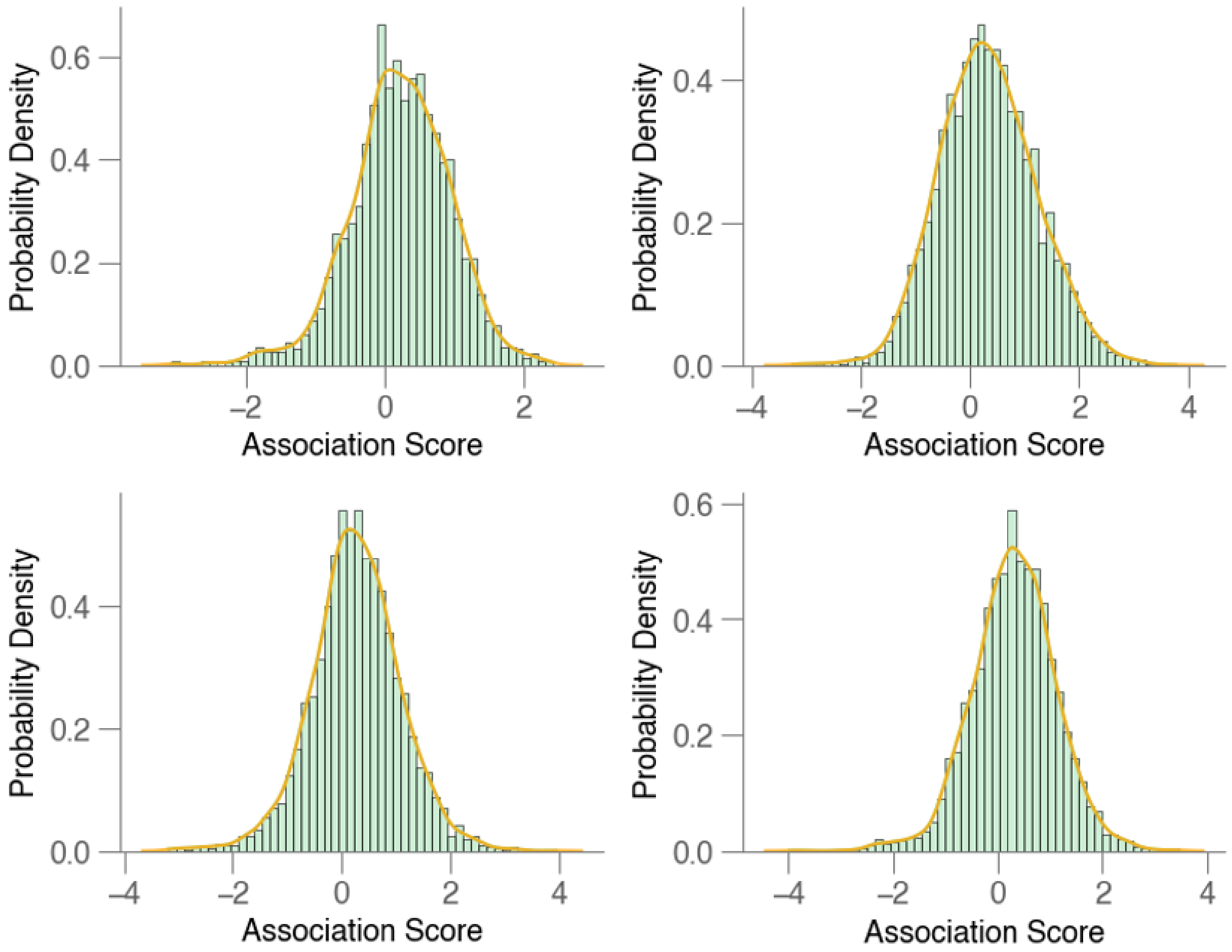

Figure S3: **Example null distributions show visual fit of KDE near normal.** Presented as an example of typical null distributions encountered when running SimPhyNI. Distributions are near normal, showcasing minor skew and/or non-zero central tendencies—features well fit by KDE. Distribution approximations are used to determine p-value and effect size for interactions.

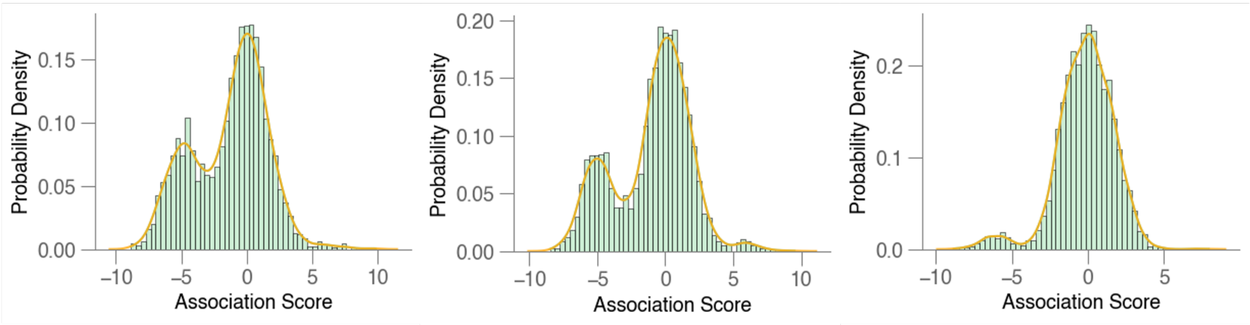

Figure S4: **Example multi-modal null distributions show visual fit of KDE for non-normal data.** Presented as an example of multi-modal null co-occurrence distributions that can arise due to a combination of topological features and trait parameters during simulation. KDE with $N = 64$ shows a visually suitable fit to these distributions, capturing non-normal behavior. Each plot is a distinct pair of synthetic traits generated using the 4-State Markov process.

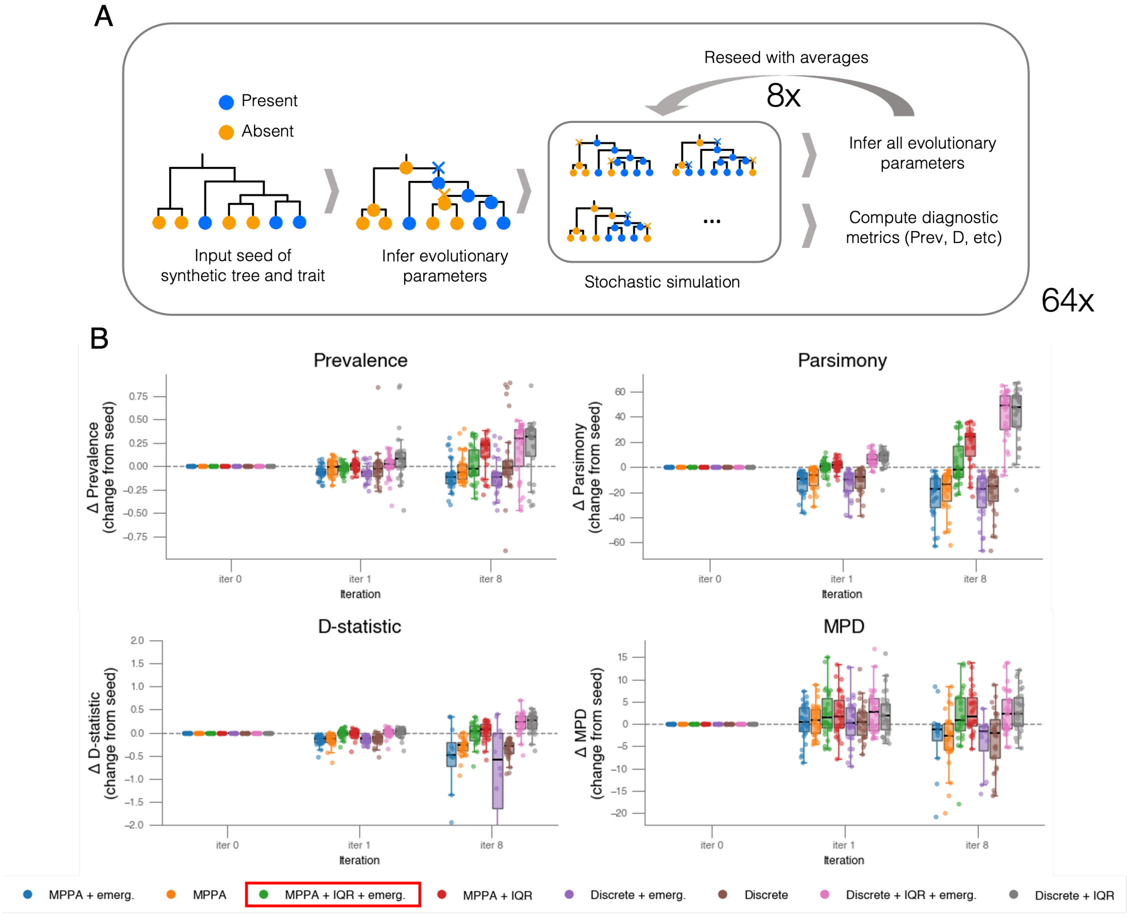

Figure S5: **Stability of trait diagnostic parameters across ACR parameterizations during iterative simulation** (A) Schematic of the iterative parameter learning and simulation. Gain and loss rates are estimated from a seed trait via ACR, used to simulate a trait distribution (Methods), and a representative simulation (selected by minimum RMSD to the mean gain and loss rates across 64 simulated traits) is used to re-seed the next iteration. Each of 64 traits was run for 8 iterations of learning and simulation. (B) Deviation of diagnostic parameters from the seed pair at iteration 1 (approximating parameters as they would be used in practice by SimPhyNI) and iteration 8 (assessing long-run drift), across eight ACR parameterizations. Panels show change from seed in prevalence (top left), Fitch parsimony (top right), D-statistic (bottom left), and MPD (bottom right). Well-calibrated parameterizations show tight distributions centered near zero with minimal directional drift across iterations; distributions are expected to widen modestly as trials accumulate but should not shift systematically. Discrete-based parameterizations show pronounced directional drift in parsimony and D-statistic by iteration 8, indicating progressive divergence from seed trait properties. MPPA-based methods remain centered near zero across all metrics with limited drift. MPD is expected to show wide distributions throughout, as simulations are stochastic by design; importantly, MPD distributions should remain stable in width across iterations rather than collapsing toward zero or expanding toward maximum prevalence — instability here would indicate degeneracy. The selected parameterization (MPPA + IQR + emerg., green; highlighted) demonstrates consistent centering, controlled spread, and stable MPD across iterations, supporting its use as the default ACR configuration.

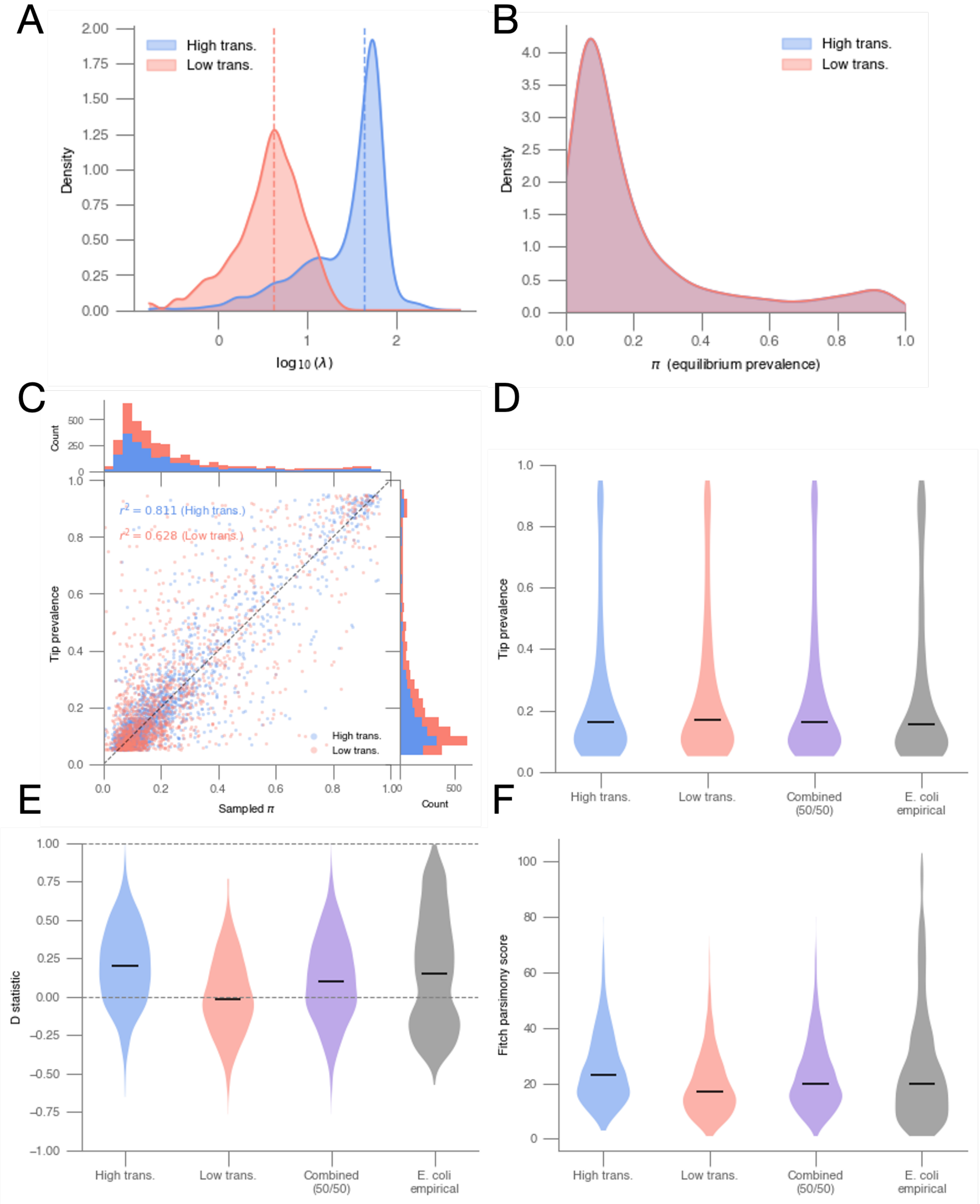

Figure S6: **Synthetic data from high and low transition models mirror central tendency and the range of *E. coli* diagnostic parameters.** (A) Empirical distributions of Λ (total transition rate), for the high transition (total marginal-state computed rates), and low transition (total discrete-state computed rates) models for all accessory genes (Methods: Section Synthetic Dataset Construction). (B) Empirical distributions of π (predicted stationary prevalence) for for the high and low transition models (Identical by design). (C) Tip prevalence of simulated genes compared with sampled π values across both transition models. The r^2 values align with the expectation that phylogenetic structure will prevent perfect alignment with computed π values and that higher transition rates result in increased convergence to the expectation. (D-F) Tip prevalence, D statistic, and Fitch parsimony of synthetic data compared to the empirical distribution of *E. coli*.

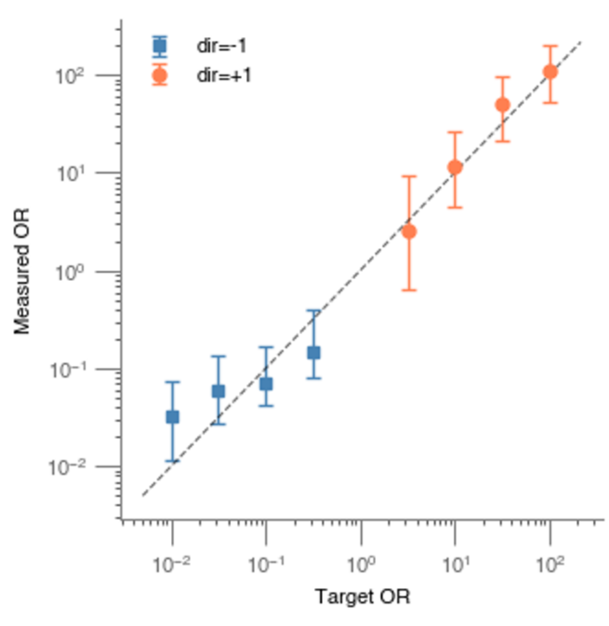

Figure S7: **Joint stationary distribution adjustment via an interaction parameter results in tunable changes to the measured OR in of synthetic trait tip co-occurrence.** Target OR reflects the expected OR from the interaction modified stationary distributions of synthetic traits. When interacting models are simulated on a phylogenetic tree, the topology prevents exact matches of target OR (Methods: section Synthetic Data Construction). Notably negative associations become saturated quickly as traits tend to have low prevalence making co-occurrence already rare

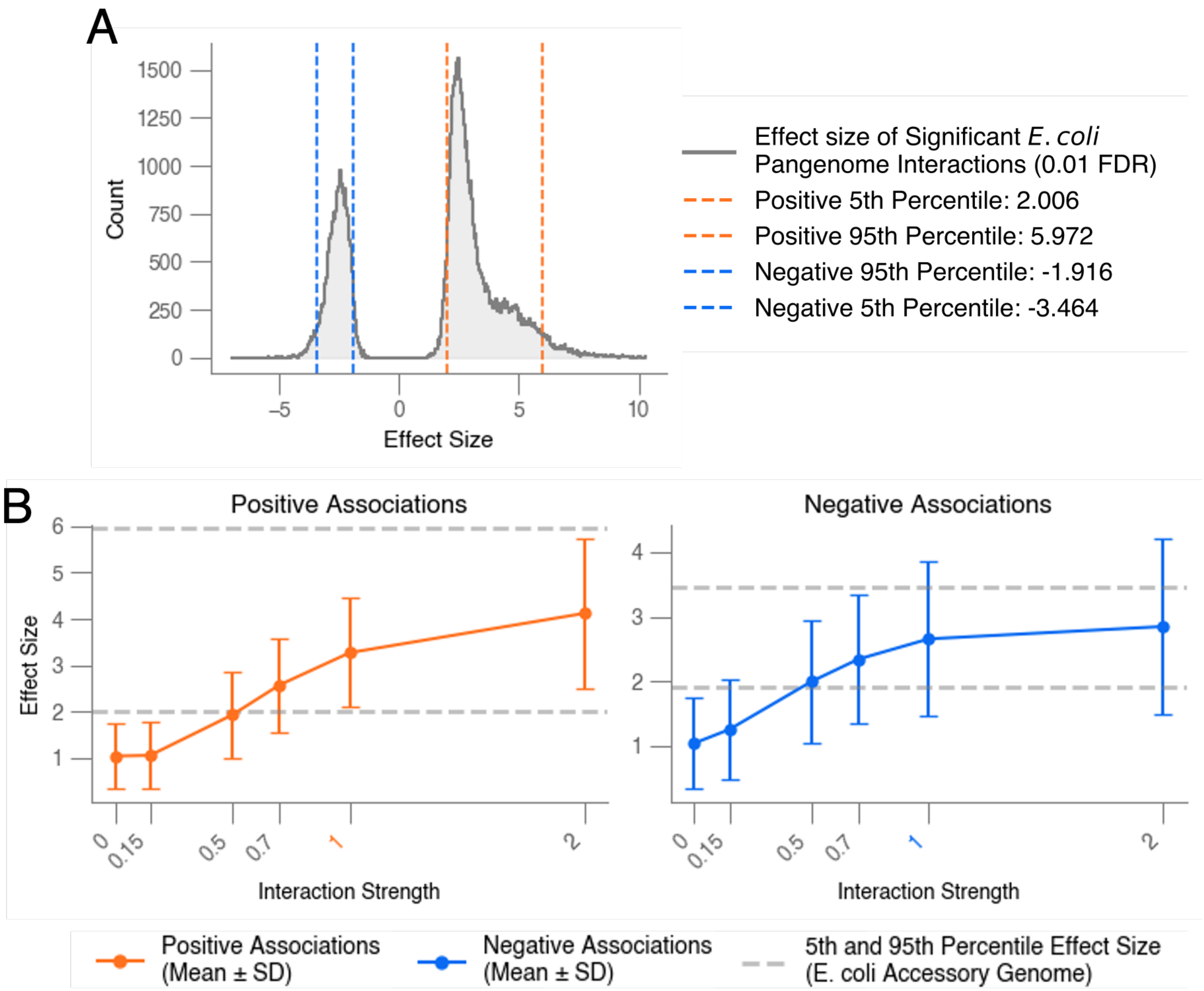

Figure S8: **Distributions and benchmarking of synthetic data input interaction strength against effect sizes from significant trait-trait associations in the *E. coli* pangenome.** (A) Histogram of effect sizes for all significant associations (FDR-BY < 0.01) identified by SimPhyNI. Dashed vertical lines indicate the 5th and 95th percentiles of effect sizes for positive (orange) and negative (blue) associations, providing empirical thresholds for interaction effect sizes. (B) Mean $\pm$ standard deviation of recovered effect sizes across varying interaction strengths in synthetic benchmarking datasets. Positive (left, orange) and negative (right, blue) association classes are shown separately. The dashed lines mark the empirical 5th and 95th percentile effect sizes from the real *E. coli* data (A) used as a reference for selecting representative interaction strengths ($I = \pm 1$) for synthetic data evaluation.

Table S3: Statistical comparisons between benchmarking metrics and SimPhyNI at interaction strength $I = \pm 1$ using two-sided Mann–Whitney U test

	**PR AUC**		**FPR**		**Precision**		**Recall**		$\mathbf{F_1}$	
**Method**	mean ± SD	p	mean ± SD	p	mean ± SD	p	mean ± SD	p	mean ± SD	p
**Positive associations ($I = +1$)**										
SimPhyNI	0.969 ± 0.006	—	0.0002 ± 0.0003	—	0.996 ± 0.005	—	0.693 ± 0.030	—	0.817 ± 0.021	—
FaST-LMM	0.621 ± 0.033	<0.001	0.0041 ± 0.0010	<0.001	0.868 ± 0.037	<0.001	0.302 ± 0.044	<0.001	0.447 ± 0.052	<0.001
TreeWAS	0.658 ± 0.016	<0.001	0.0002 ± 0.0002	0.834	0.994 ± 0.005	0.270	0.387 ± 0.023	<0.001	0.557 ± 0.024	<0.001
Pagel	0.668 ± 0.038	<0.001	0.0007 ± 0.0005	0.045	0.985 ± 0.011	0.003	0.532 ± 0.038	<0.001	0.690 ± 0.032	<0.001
Scoary	0.914 ± 0.015	<0.001	0.0000 ± 0.0000	0.016	0.000 ± 0.000	<0.001	0.000 ± 0.000	<0.001	0.000 ± 0.000	<0.001
Coinfinder	0.827 ± 0.023	<0.001	0.0318 ± 0.0050	<0.001	0.698 ± 0.030	<0.001	0.803 ± 0.032	<0.001	0.746 ± 0.019	<0.001
Fisher's	0.906 ± 0.015	<0.001	0.0739 ± 0.0075	<0.001	0.535 ± 0.023	<0.001	0.933 ± 0.016	<0.001	0.680 ± 0.019	<0.001
**Negative associations ($I = -1$)**										
SimPhyNI	0.882 ± 0.016	—	0.0002 ± 0.0002	—	0.994 ± 0.005	—	0.406 ± 0.057	—	0.575 ± 0.059	—
FaST-LMM	0.324 ± 0.041	<0.001	0.0036 ± 0.0014	<0.001	0.612 ± 0.128	<0.001	0.063 ± 0.020	<0.001	0.114 ± 0.035	<0.001
TreeWAS	0.255 ± 0.025	<0.001	0.0000 ± 0.0001	0.006	0.996 ± 0.014	0.027	0.084 ± 0.014	<0.001	0.154 ± 0.023	<0.001
Pagel	0.425 ± 0.041	<0.001	0.0134 ± 0.0020	<0.001	0.642 ± 0.042	<0.001	0.264 ± 0.032	<0.001	0.373 ± 0.037	<0.001
Scoary	0.695 ± 0.027	<0.001	0.0000 ± 0.0000	0.003	0.000 ± 0.000	<0.001	0.000 ± 0.000	<0.001	0.000 ± 0.000	<0.001
Coinfinder	0.490 ± 0.029	<0.001	0.0181 ± 0.0032	<0.001	0.651 ± 0.043	<0.001	0.368 ± 0.030	0.049	0.469 ± 0.031	<0.001
Fisher's	0.664 ± 0.027	<0.001	0.0625 ± 0.0066	<0.001	0.493 ± 0.027	<0.001	0.666 ± 0.029	<0.001	0.566 ± 0.023	0.743

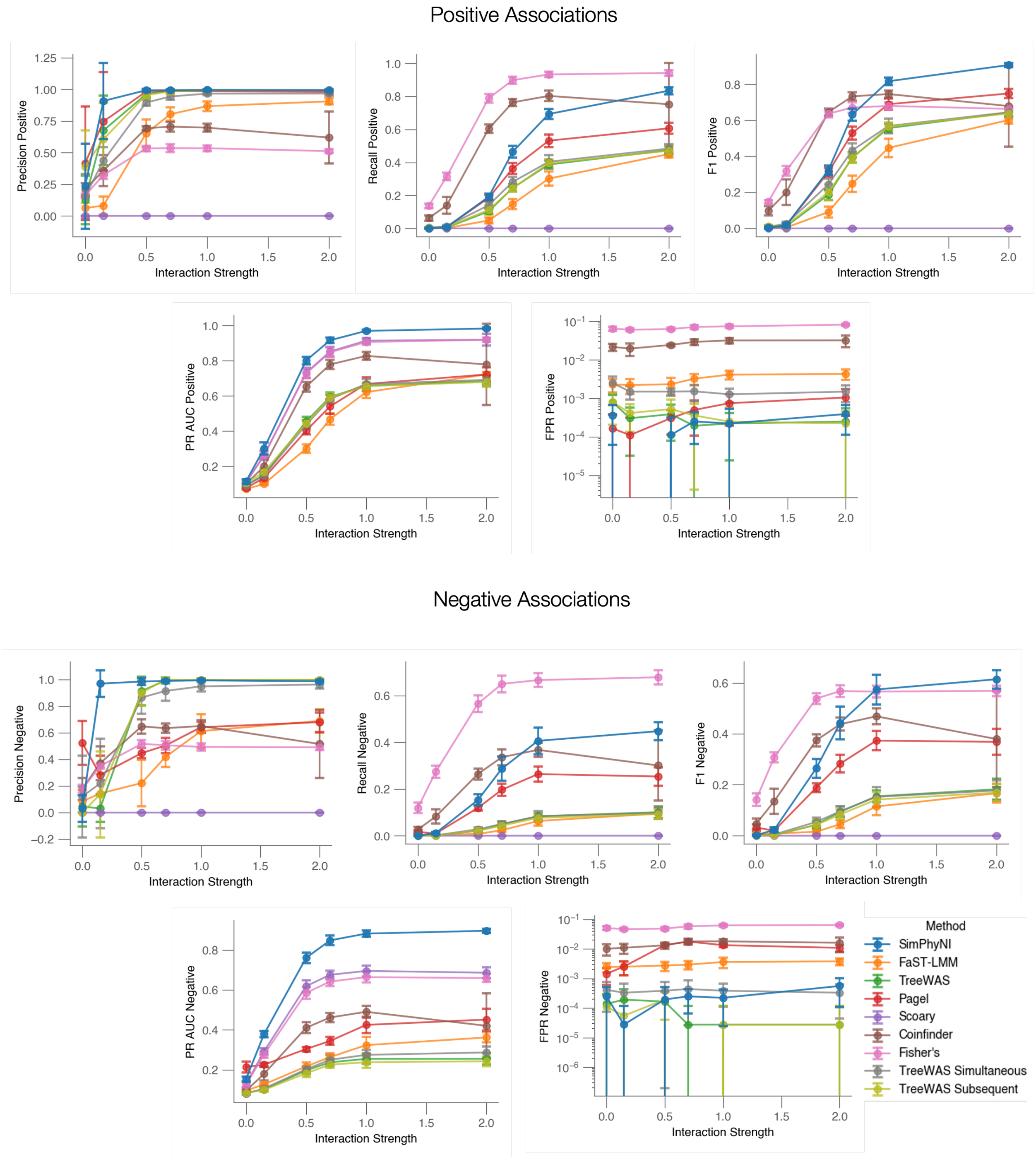

Figure S9: **All metrics for all tested methods using 4-state Markov data generation across all tested interaction strengths.** Benchmarking of all tested methods using synthetic datasets and evaluated using a Benjamini-Yekutieli multiple testing correction at FDR = 0.01. Points show mean ± standard deviation for 11 distinct phylogenetic trees at the given interaction strength. Five metrics are shown: precision, recall, F1-score, PR AUC, and false positive rate (FPR), each for both positive and negative association classes. SimPhyNI outperforms other tools across most metrics, achieving better performance at lower interaction strengths and converging to superior values as interaction strength increases.

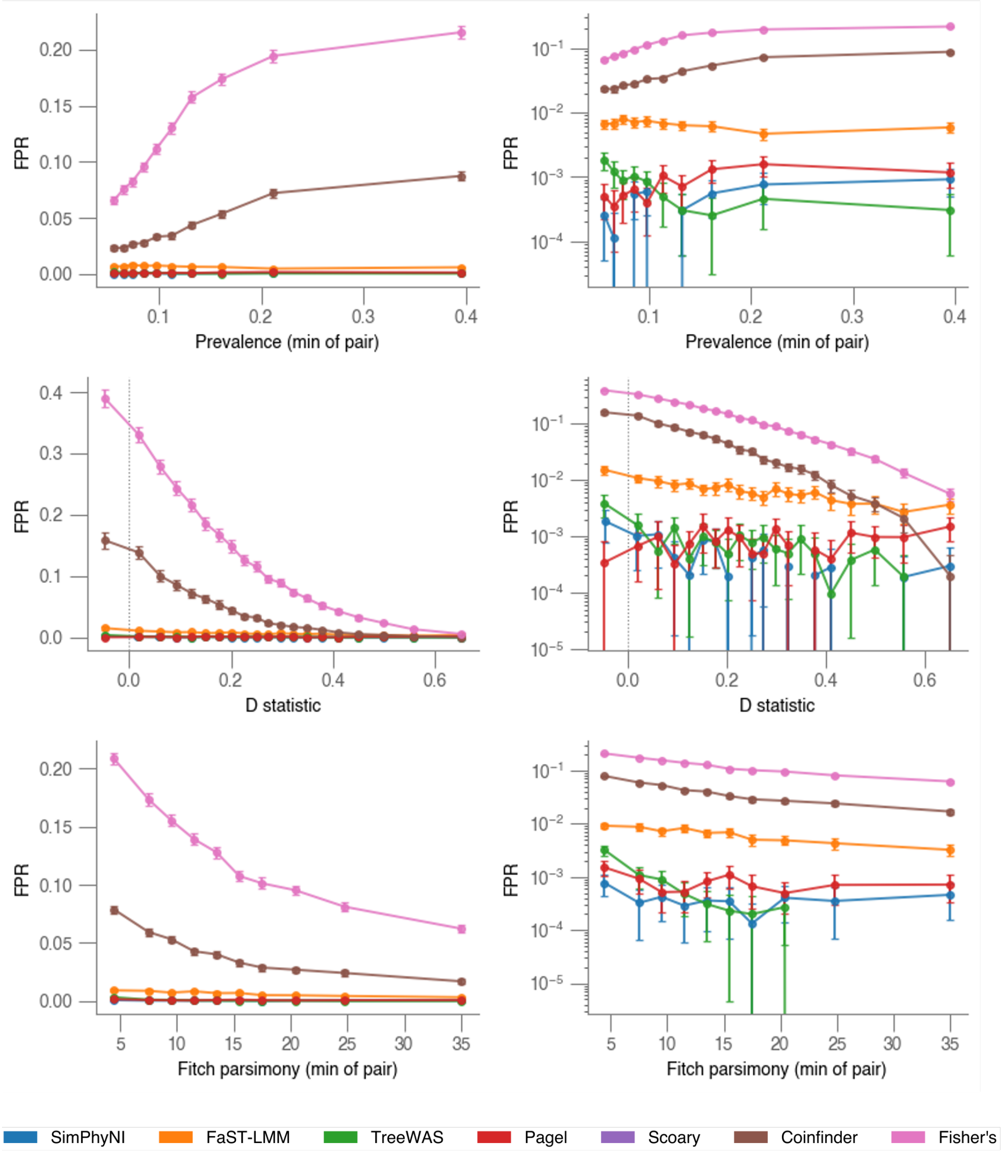

Figure S10: **False positive rate stratified by trait prevalence, phylogenetic signal, and parsimony score.** FPR for each method stratified into bins (minimum 20 trait pairs) by pairwise minimum prevalence (top), maximum phylogenetic D-statistic (middle), and minimum Fitch parsimony score (bottom). Left panels show linear scale; right panels show the same data on a log scale to resolve differences among low-FPR methods. Points represent binned means across 11 synthetic datasets. The vertical dashed line marks $D = 0$ representing Brownian motion like assortment. Fisher's exact test shows strongly inflated FPR that increases with prevalence and decreases with D and parsimony score, consistent with failure to account for phylogenetic non-independence.

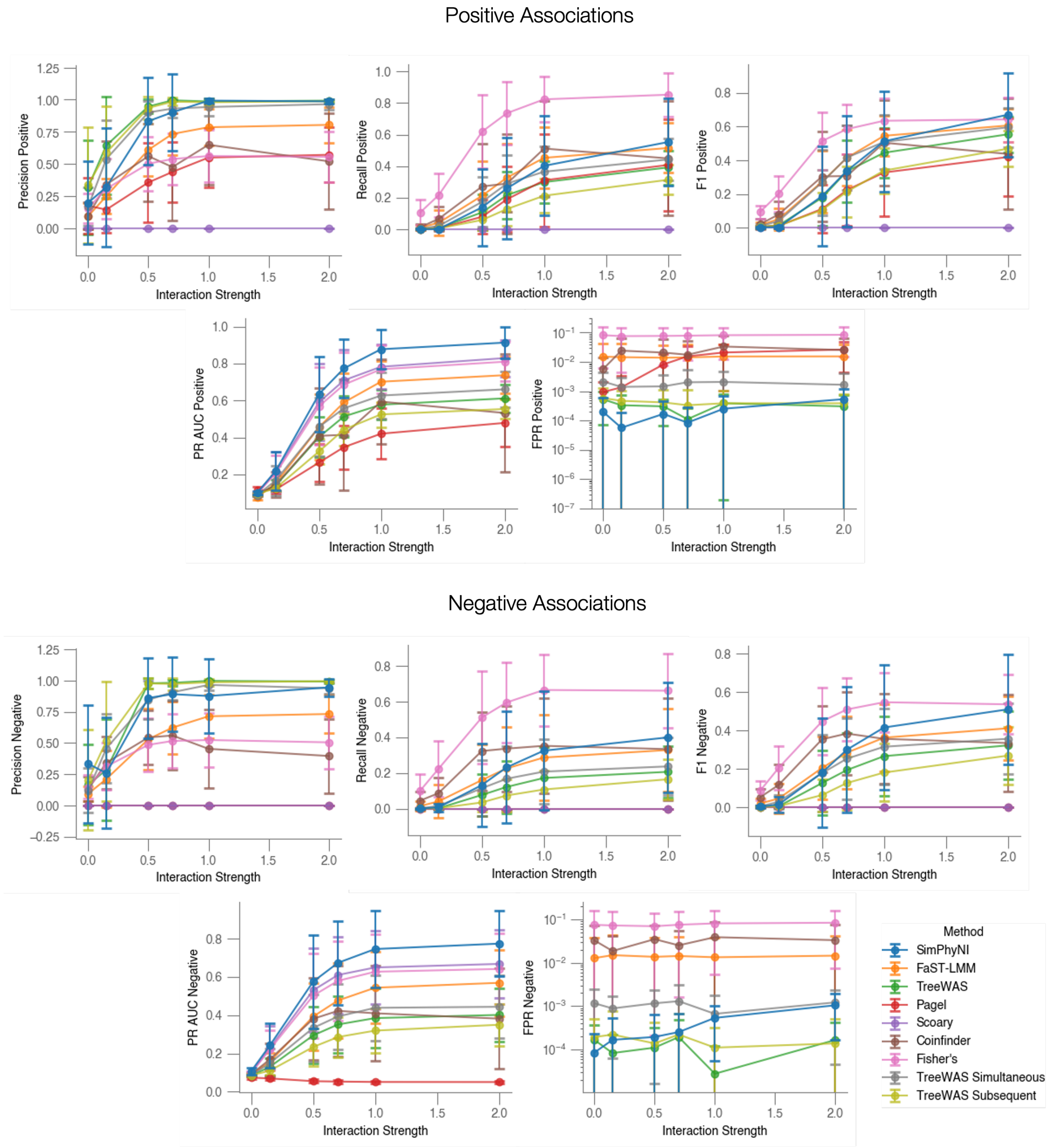

Figure S11: **Method performance on synthetic data generated using real bacterial species trees across a range of interaction strengths.** Performance metrics for all benchmarked methods evaluated on synthetic interacting trait pairs generated using 11 bacterial phylogenetic trees from the PanX database (Methods: Section Benchmarking Against Other Methods). Each panel shows mean performance averaged over all 11 species trees at each tested interaction strength; error bars denote one standard deviation across trees. SimPhyNI maintains superior PR AUC and near-zero FPR across all species trees tested, consistent with primary benchmarking results (Figure S9) Variance in performance across trees is driven primarily by effective sample size: smaller trees yield less extreme realized co-occurrence for simulated trait pairs, reducing statistical power uniformly across methods.

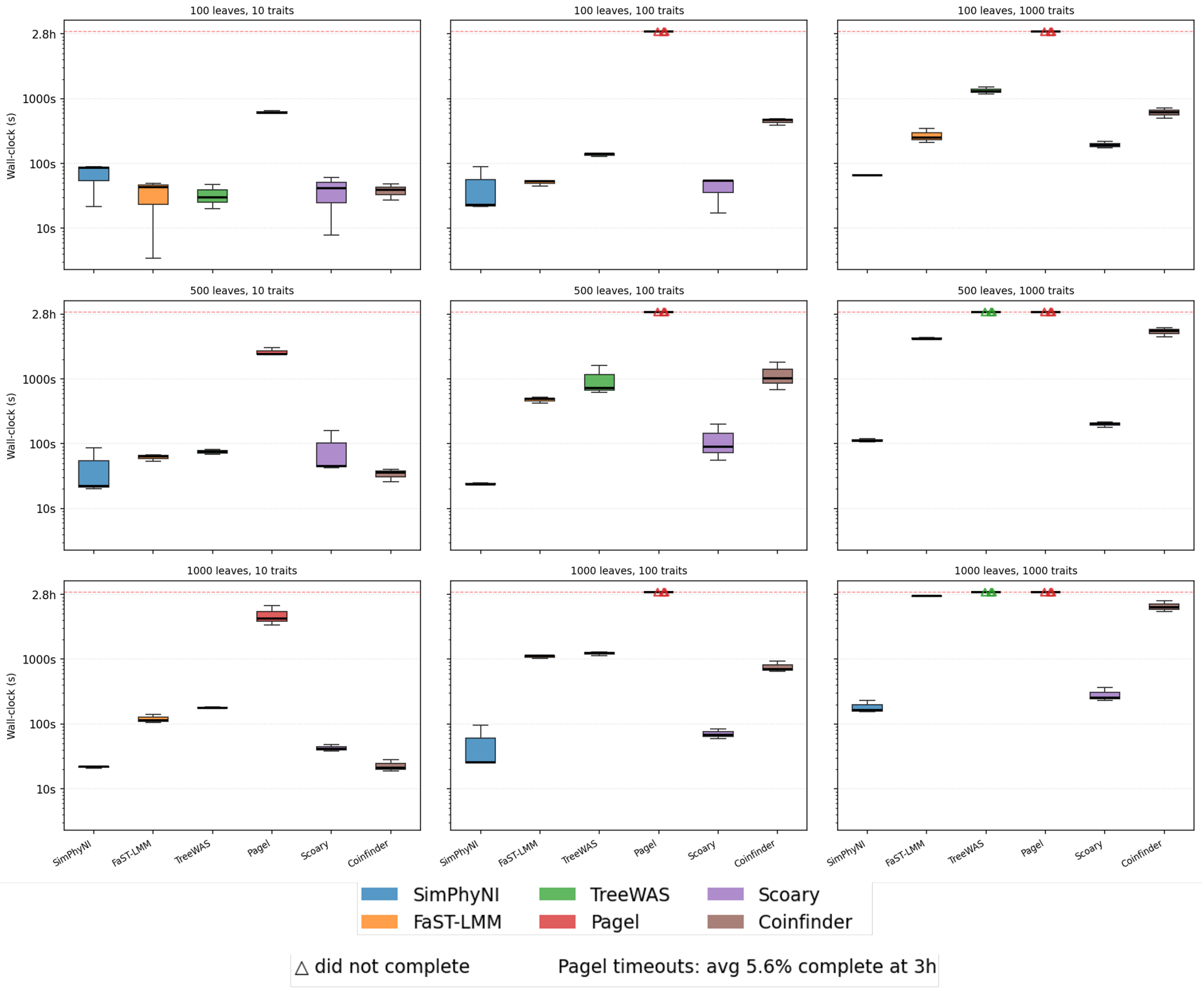

Figure S12: **Runtime of SimPhyNI and other tested methods for all against all comparisons on various dataset sizes.** Runtime in seconds for each method on 27 synthetic datasets of different tree sizes and trait counts. Tools were used to test interactions for all combinations of traits parallelized across 16 CPU cores, with total 64 GB RAM. A time limit of 3 hours was set for analyses with TreeWAS and Pagel's correlation method reaching this limit on larger datasets. SimPhyNI uses JIT compilation of key functions to machine code to rapidly perform ancestral character reconstruction and null model computation, requiring one system-wide compile and function cache; this is not shown here but it takes 10-100 seconds. Notably, parallelization is not a direct part of TreeWAS so the mcapply R function was used across 4 cores with time limit of 12 hours and times were scaled accordingly.

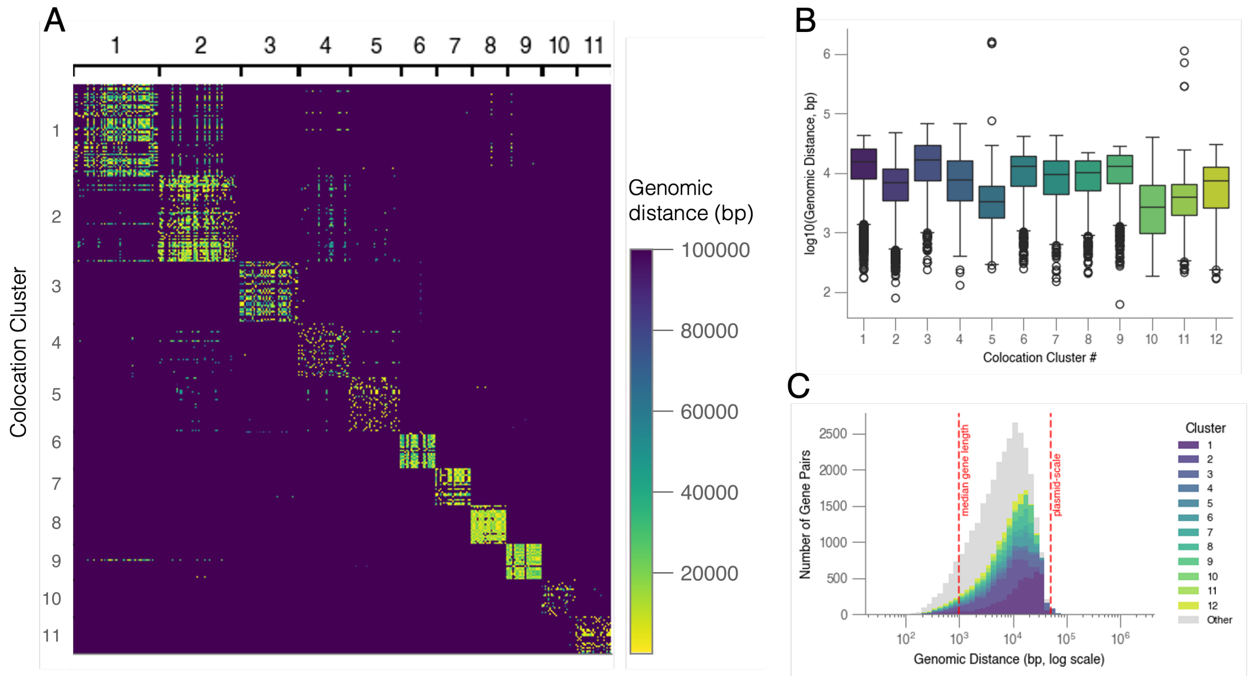

Figure S13: **Genomic distances within co-location clusters made from *E. coli* accessory genes.** (A) A heatmap of genomic distance between genes in the top 11 largest co-location clusters. (B) Distribution of pairwise distances for the top 11 co-location clusters. (C) Within cluster pairwise distances for all co-location clusters. Contributions of top 11 co-location clusters are labeled and reference points for gene and plasmid scale is annotated.

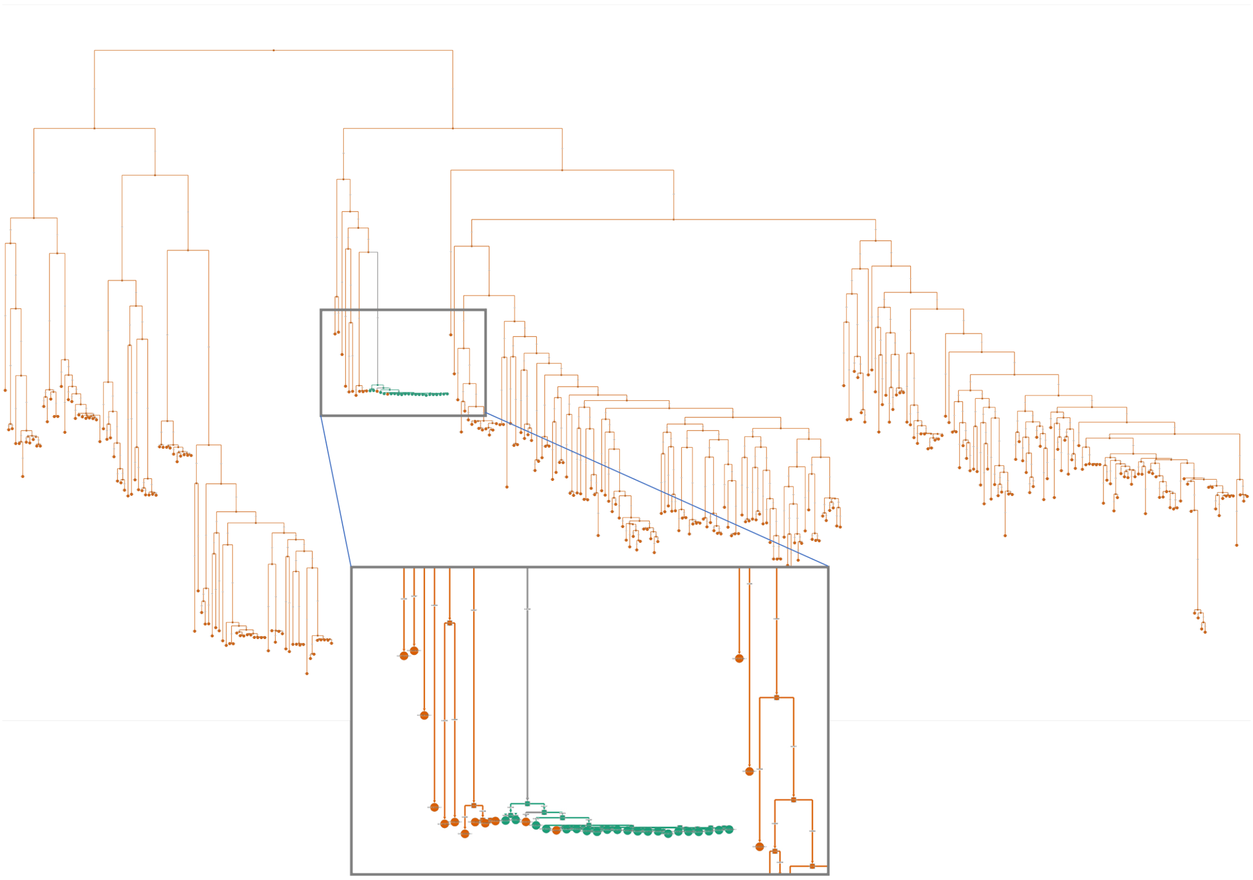

Figure S14: **Poorly resolved ordering in outbreak-like clusters on the phylogenetic tree creates spurious associations.** A clonal expansion on phylogenetic tree in which multiple events are inferred to occur. Due to near zero genomic distance between strains, the correct ordering of strains becomes ambiguous. Further this outbreak contains the only occurrence of this gene, giving inaccurate transition rates for the gene, introducing spurious associations.

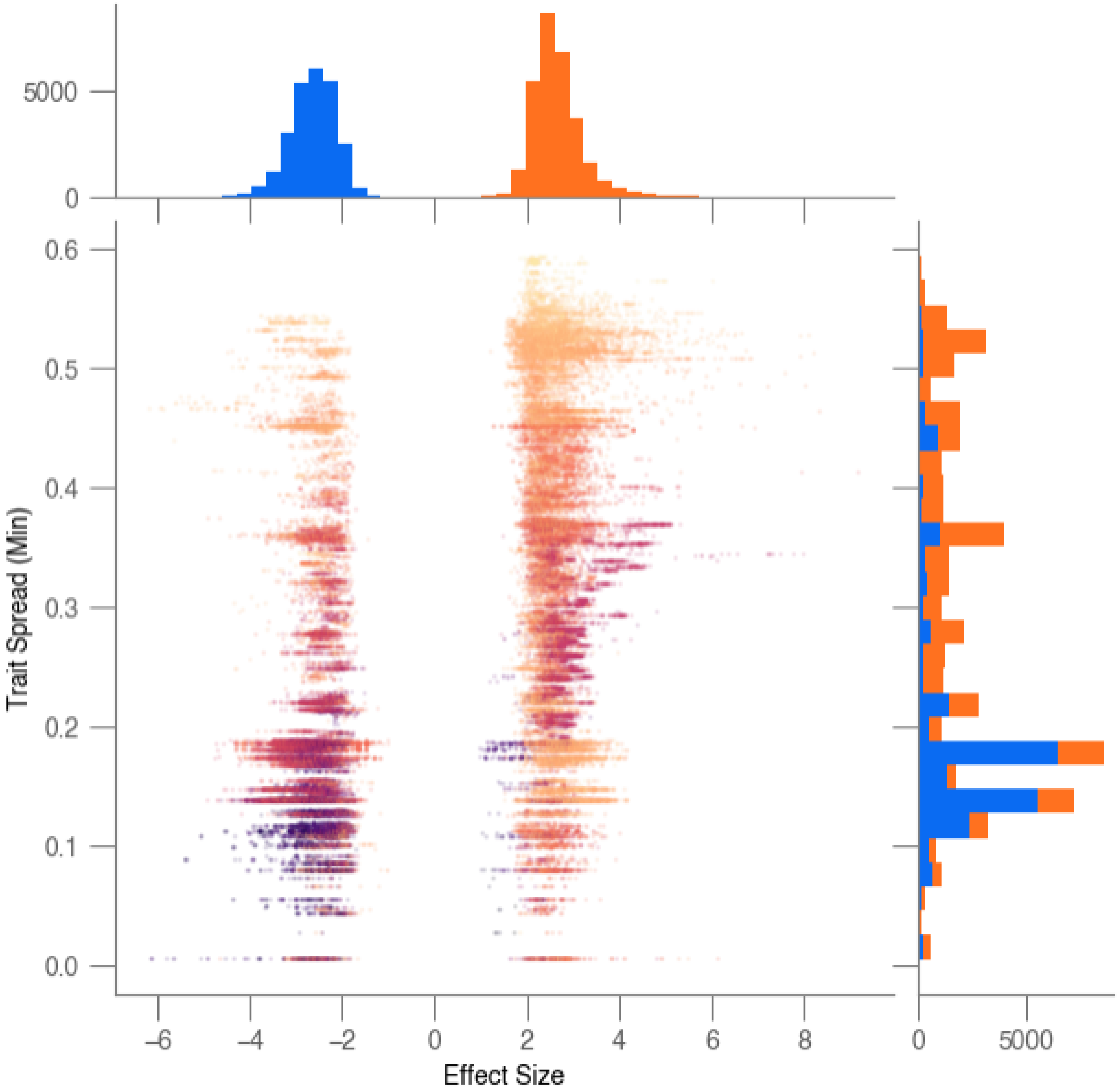

Figure S15: **Distributions of phylogenetic spread compared against effect size of significant interactions between *E. coli* accessory genes.** A scatter plot of significant interactions (Benjamini-Yekutieli < 0.01) filtered to remove co-location derived interactions, visualizing their effect size and phylogenetic spread. Position of points is determined by the minimum spread of interacting traits while color is determined by the maximum. Marginals show counts along each axis colored by the direction of the interaction.

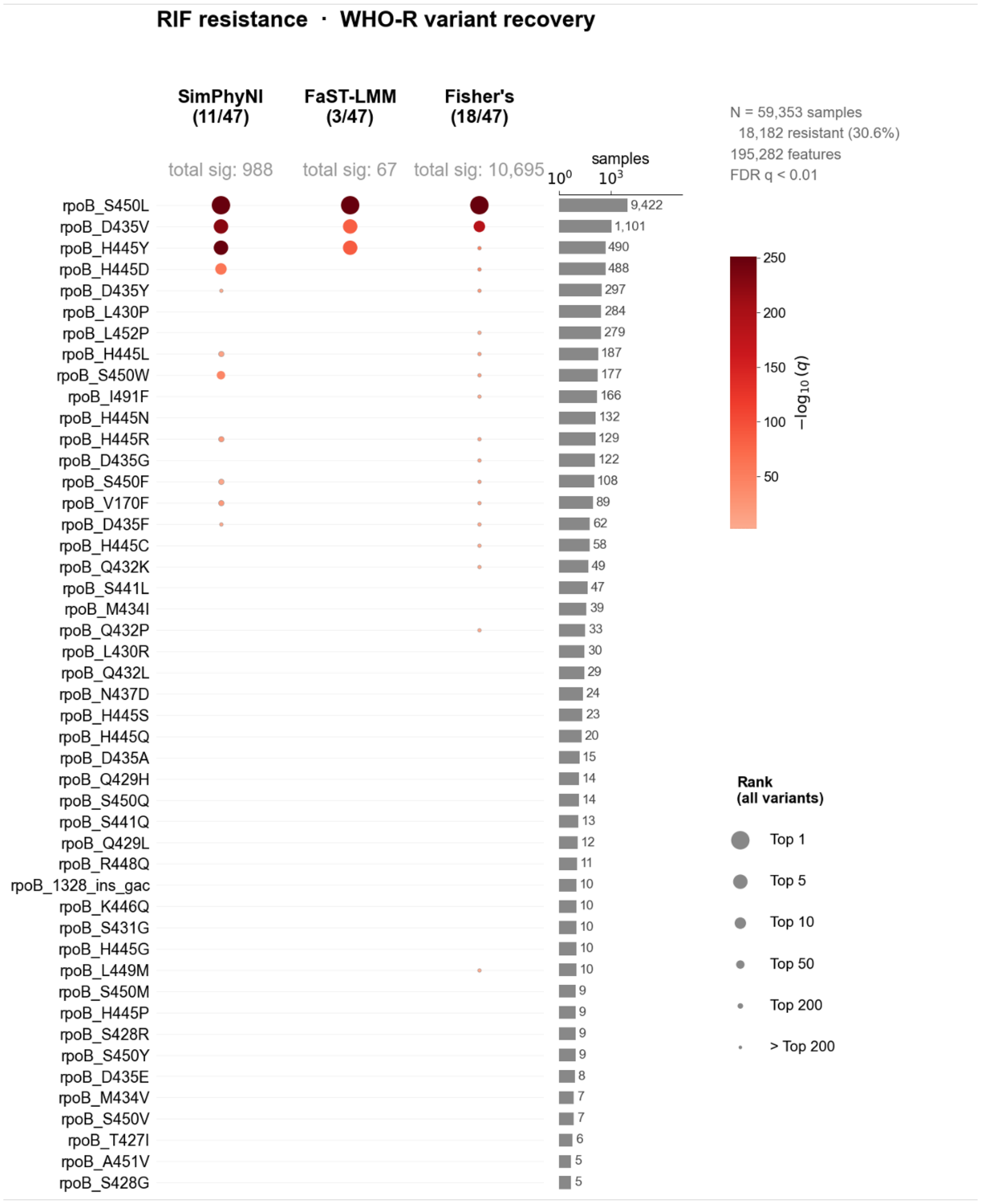

Figure S16: **SimPhyNI scales to large sample and feature spaces, recovering and ranking known causal SNVs for rifampin resistance in *Mycobacterium tuberculosis*.** The three methods, chosen for their efficiency, scalability, and ability to take large input feature sets, were applied to the CRyPTIC project MTB dataset, testing genome wide variants (SNVs, indels) for associations with RIF resistance (Methods: Section Analysis of Mycobacterium tuberculosis Rifampicin Resistance Mutations). All methods show ranking that correlates variant frequency in the dataset, with low frequency variants being undetectable by all methods. SimPhyNI shows excellent performance, recovering more mutants than FaST-LMM with high rankings while approaching the recall Fisher's exact test with only a fraction ($< 10\%$) of the total significant hits. We note that there are significantly associated biologically meaningful non-casual variants (e.g. compensatory mutations in rpoC and rpoA for fitness and co-carried resistant mutations in multi-drug resistant MTB); however, in lieu of rigorous annotation, we report results in Table S6
